# Effect of Temperature on Photoisomerization Dynamics of a Newly Designed Two-Stroke Light-Driven Molecular Rotary Motor

**DOI:** 10.3390/ijms23179694

**Published:** 2022-08-26

**Authors:** Jianzheng Ma, Di Zhao, Chenwei Jiang, Zhenggang Lan, Fuli Li

**Affiliations:** 1 Ministry of Education Key Laboratory for Nonequilibrium Synthesis and Modulation of Condensed Matter, Shaanxi Province Key Laboratory of Quantum Information and Quantum Optoelectronic Devices, School of Physics, Xi’an Jiaotong University, Xi’an 710049, China; 2 Guangdong Provincial Key Laboratory of Chemical Pollution and Environmental Safety & MOE Key Laboratory of Environmental Theoretical Chemistry, SCNU Environmental Research Institute, School of Environment, South China Normal University, Guangzhou 510006, China

**Keywords:** unidirectionality, quantum yield, photon-only, trajectory surface-hopping simulation, temperature

## Abstract

The working mechanism of conventional light-driven molecular rotary motors, especially Feringa-type motors, contains two photoisomerization steps and two thermal helix inversion steps. Due to the existence of a thermal helix inversion step, both the ability to work at lower temperatures and the rotation speed are limited. In this work, a two-stroke light-driven molecular rotary motor, 2-(1,5-dimethyl-4,5-dihydrocyclopenta[b]pyrrol-6(1H)-ylidene)-1,2-dihydro-3H-pyrrol-3-one (DDPY), is proposed, which is capable of performing unidirectional and repetitive rotation by only two photoisomerization (*EP→ZP* and *ZP→EP*) steps. With trajectory surface-hopping simulation at the semi-empirical OM2/MRCI level, the *EP*→*ZP* and *ZP→EP* nonadiabatic dynamics of DDPY were systematically studied at different temperatures. Both *EP→ZP* and *ZP→EP* photoisomerizations are on an ultrafast timescale (ca. 200–300 fs). The decay mode of *EP*→*ZP* photoisomerization is approximately bi-exponential, while that of *ZP→EP* photoisomerization is found to be periodic. For *EP* and *ZP* isomers of DDPY, after the S${}_{0}$→S${}_{1}$ excitation, the dynamical processes of nonadiabatic decay are both followed by twisting about the central C=C double bond and the pyramidalization of the C atom at the stator-axle linkage. The effect of temperature on the nonadiabatic dynamics of *EP*→*ZP* and *ZP→EP* photoisomerizations of DDPY has been systematically investigated. The average lifetimes of the S${}_{1}$ excited state and quantum yields for both *EP*→*ZP* and *ZP→EP* photoisomerization are almost temperature-independent, while the corresponding unidirectionality of rotation is significantly increased (e.g., 74% for *EP*→*ZP* and 72% for *ZP→EP* at 300 K *vs* 100% for *EP*→*ZP* and 94% for *ZP→EP* at 50 K) with lowering the temperature.

## 1. Introduction

Molecular machines [1,2] are a kind of nanomachine that can perform controllable and continuous changes of molecular structure under the stimulation of external energy source, so as to complete specific tasks. Such archetypal machines are molecular motors [3,4,5,6,7,8] which are capable of performing unidirectional and repetitive rotation at the nanoscale upon the stimulation of external energy. Light-driven molecular rotary motors (LDMRMs) [2,5,6], which can utilize the photoinduced *E-Z* (or *cis-trans*) isomerization of the double bond (to date, carbon-carbon double bond [9,10,11,12,13,14,15] or carbon-nitrogen double bond [16,17,18]) to complete a full 360${}^{\circ }$ rotation by the absorption of UV or visible light, have attracted considerable interest in recent decades because of the high efficiency and cleanliness.

The complete 360${}^{\circ }$ rotation of conventional LDMRMs, especially Feringa-type motors [9,10,11,12,13], is achieved through two steps of photoisomerization combined with two steps of thermal helix inversion (THI). The timescale of the THI steps (from nanoseconds to days) is much longer than that of photoisomerization steps (from femtoseconds to picoseconds) [6]. Thus, reducing the THI steps can not only improve the rotation speed but also enable LDMRM to operate at lower temperatures [6,19,20]. Some developments have been achieved on reducing the THI steps theoretically [21,22,23,24,25] and experimentally [15,26] in recent years.

From a theoretical aspect, by generating a chiral hydrogen bond environment, a two-stroke LDMRM which involves only two steps of photoisomerization was proposed by García-Iriepa et al. [21]. By redesigning a bio-inspired 4-hydroxybenzylidene-1,2-dimethylimidazolinone-based molecular photoswitch [27], Filatov et al. [22] proposed a family of two-stroke photon-only LDMRMs recently. With the nonadiabatic molecular dynamics (NAMD) simulations at the SSR-BH&HLYP/6-31G(d) level, these molecular motors are predicted to have very high quantum yields (about 0.91–0.97) and a sufficiently high degree (0.94–1.00) of unidirectionality. A visible-light responsive Schiff-based LDMRM, which is able to complete a 360${}^{\circ }$ unidirectional rotation by only two photoisomerization steps, was designed by Wang et al. [23] recently. The quantum yields were predicted to be almost 70% for its individual *E* to *Z* and *Z* to *E* photoisomerizations using NAMD simulations. A novel molecular motor in which the rotation is induced by the electric coupling of chromophores was suggested by Majumdar et al. [24] recently, which was predicted to achieve unidirectional rotation on a subnanosecond time scale using the power of a single photon. A three-stroke LDMRM, 2-(2,7-dimethyl-2,3-dihydro-1Hinden-1-ylidene)-1,2-dihydro-3H-pyrro- l-3-one (DDIY), was proposed by our group [25] very recently, which is capable of completing a unidirectional rotation by two photoisomerization steps and one thermal helix inversion step at room temperature.

On the experimental side, Gerwien et al. [15] designed a three-stroke photon-only hemithi-oindigo-based molecular motor recently, which interconverts three different isomeric states in a fixed sequence upon visible light irradiation, without thermal ratcheting in the ground state. Three new second-generation molecular motors featuring a phosphorus center in the lower half have been reported by Boursalian et al. [26] recently. Four diastereomeric states of these molecular motors can interconvert solely photochemically. All-photochemical unidirectional rotation of the new molecular motors was confirmed by kinetic analysis and modeling.

Although some excellent developments on photon-only molecular motors [21,22,23] have been achieved from a computational perspective, Feringa et al. [6] pointed out very recently that synthesizing of these molecular motors in experiments is often highly challenging. Designing a light-driven molecular motor with fewer operational steps based on synthesized molecular systems may be an effective way. Based on some easy-to-synthesize oxindole-based molecular motors studied by Roke et al. [12] and Pooler et al. [13] recently, by further reducing the steric hindrance in the fjord region of the LDMRM DDIY proposed in our previous work [25], a new two-stroke LDMRM, 2-(1,5-dimethyl-4,5-dihydrocyclopenta[b]pyrrol-6(1H)-ylidene)-1,2-dihydro-3H-pyrrol-3-one (DDPY), was designed, in which only two photoisomerization steps (*EP*→*ZP* and *ZP→EP*) are involved to complete a full 360${}^{\circ }$ rotation. The photoinduced isomerization dynamics of this two-stroke LDMRM were systematically investigated with trajectory surface-hopping molecular dynamics at the semi-empirical OM2/MRCI level.

It is well known that temperature plays a very crucial role in chemical reactions, e.g., affecting the rate and direction of a chemical reaction. The effect of temperature on the thermal helix inversion steps of LDMRMs has been extensively studied [5,6]. However, to the best of our knowledge, much less is known about the effect of temperature on the photoisomerization processes of LDMRMs, especially the unidirectionality [28] of LDMRMs. Very recently, Gerwien et al. [15] found that the monodirectionality [28] and quantum yield increase with lowering the temperature for a three-step photon-only LDMRM. To figure out the effect of temperature on photoisomerization dynamics of LDMRM DDPY, non-adiabatic dynamics simulations at different temperatures were systemically performed. The dynamic results show that, as the temperature decreases, both the average S${}_{1}$ lifetime and quantum yield of *EP*→*ZP* and *ZP→EP* photoisomerization processes of LDMRM DDPY are almost temperature-independent, while the corresponding unidirectionality increases significantly, e.g., 74% for *EP*→*ZP* and 72% for *ZP→EP* at 300 K vs. 100% for *EP*→*ZP* and 94% for *ZP→EP* at 50 K.

## 2. Methods and Materials

### 2.1. Density Functional Methods

The geometrical optimization and frequency calculation of ground state and transition state of molecular motor DDPY by density functional theory (DFT) were performed at the B3LYP/6-31G(d), CAM-B3LYP/6-31G(d), and B3LYP-D3/6-31+G(d) levels. The GAUSSIAN 09 program [29] was utilized to carry out all the DFT calculations.

### 2.2. Semiempirical Methods

The OM2/MRCI method implemented in the development version of the MNDO program [30] was utilized to perform all the semi-empirical calculations. This method can balance the computational cost and accuracy well, as confirmed by many benchmark calculations [31,32,33], and has been applied to investigate many photoinduced processes [34,35,36,37,38,39,40,41,42,43,44,45] successfully.

For geometry optimizations and dynamics simulations, all required energies, gradients, and nonadiabatic coupling elements were computed analytically. The self-consistent field (SCF) calculations were performed in the restricted open-shell Hartree–Fock (ROHF) formalism, as it provided a better description of the excited-state wave functions. For the multireference configuration interaction (MRCI) treatment, three reference configurations were chosen, which includes the closed-shell ground-state configuration and single and double excitations from the highest occupied molecular orbital (HOMO) to the lowest unoccupied molecular orbital (LUMO). The active space in the MRCI calculations included 10 electrons in nine orbitals, which comprises four highest doubly occupied orbitals, two singly occupied orbitals, and three lowest unoccupied orbitals. The Lagrangian-Newton approach [46] was used to locate the S${}_{1}$/S${}_{0}$ minimum-energy conical intersections (CIs) geometries.

The nonadiabatic photoisomerization dynamics of molecular motor DDPY were investigated in the gas phase by the trajectory surface-hopping (TSH) simulations with Tully’s fewest-switches algorithm [47,48,49,50,51]. The initial structures and velocities for the nonadiabatic dynamics simulations were selected randomly from a 5 ps ground state trajectory at specific temperature, and then chosen using the filtering procedure implemented in the MNDO program [30] according to the computed S${}_{0}$-S${}_{1}$ transition probabilities. An empirical decoherence correction (0.1 a.u.) suggested by Granucci et al. [52] was employed. A constant time step of 0.1 fs was chosen to solve the nuclear motion equation, while a 100 times smaller time step was selected for the time-dependent electronic propagation.

## 3. Results and Discussion

### 3.1. Equilibrium Structures

**Figure 1:**
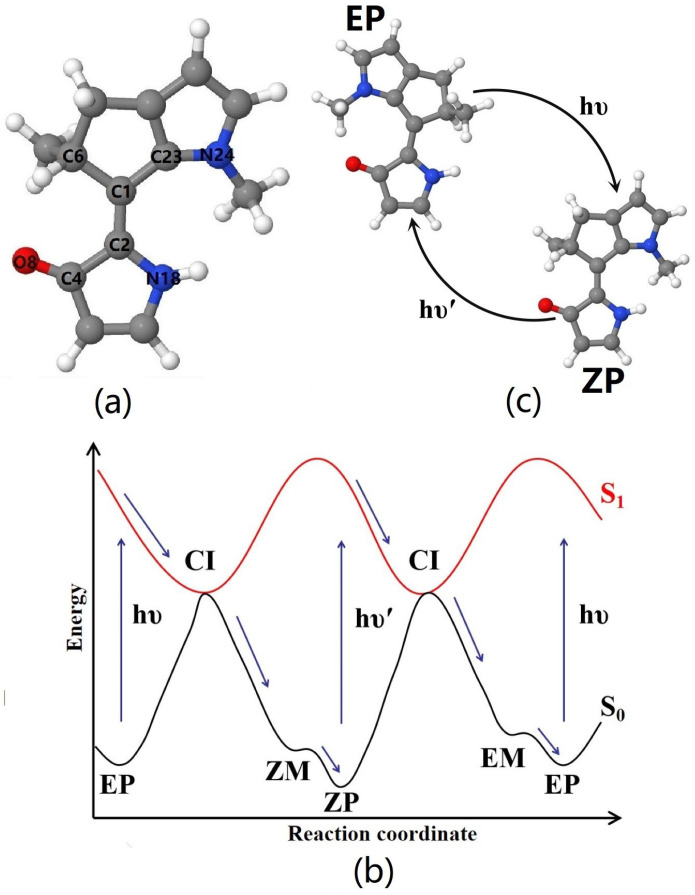
(**a**) Optimized geometry of *ZP* isomer of DDPY. Some atoms around the central C=C double bond are labeled; (**b**) the schematic diagram of ground and the first excited state potential energy profiles along the reaction coordinate of LDMRM DDPY; (**c**) the schematic diagram of a working cycle of the photon-only two-stroke LDMRM DDPY.

Four local minima geometries of DDPY in the ground state were obtained based on the OM2/MRCI, B3LYP/6-31G(d), CAM-B3LYP/6-31G(d), and B3LYP-D3/6-31+G(d) level of theories. According to the conformation and helicity, these four equilibrium structures are called *EP*, *EM*, *ZP*, and *ZM*, respectively. The approach of helicity definition proposed by Karnik et al. [53] was adopted. Geometry of the most stable isomer *ZP*-DDPY is presented in Figure 1a, while geometries of the other three isomers are shown in Figure S1 (see ESI†). The corresponding geometrical parameters of the four isomers are listed in Table S1 (see ESI†). As can be seen, the optimized geometries obtained from the different theoretical methods above are consistent with each other.

The transition state of *EM-EP-TS* (between *EM* and *EP* isomers) and *ZM-ZP-TS* (between *ZM* and *ZP* isomers) in the ground state were also optimized at the OM2/MRCI, B3LYP/6-31G(d), CAM-B3LYP/6-31G(d), and B3LYP-D3/6-31+G(d) levels. The optimized transition state geometries obtained with the OM2/MRCI method are presented in Figure S2 (see ESI†), while the corresponding geometrical parameters are summarized in Table S2 (see ESI†). As can be seen, these geometrical parameters obtained from the different methods above are in good agreement with each other. According to the obtained transition state, the energy barriers from *EM* to *EP* isomers and from *ZM* to *ZP* isomers were calculated based on the OM2/MRCI, B3LYP/6-31G(d), CAM-B3LYP/6-31G(d), and B3LYP-D3/6-31+G(d) methods, as shown in Table 1. As we can see, both energy barriers from *EM* to *EP* and from *ZM* to *ZP* isomers are very low regardless of the method, e.g., just about 0.31 kcal/mol from *EM* to *EP* and 0.47 kcal/mol from *ZM* to *ZP* obtained from the B3LYP-D3/6-31+G(d) calculations.

The schematic diagram of S${}_{0}$ and S${}_{1}$ potential energy profiles along the reaction coordinate is shown in Figure 1b. As we can see, after the S${}_{0}$→S${}_{1}$ optical excitation of *EP* or *ZP* isomer, molecular motor DDPY rotates around the central C=C double bond in a counterclockwise direction, and relaxes to the S${}_{0}$ state through the S${}_{1}$/S${}_{0}$ conical intersections (CIs), then it arrives at the metastable *ZM* or *EM* isomer. Motivated by our previous work [25] and Filatov’s work [22], due to the very low energy barriers from *ZM* to *ZP* and from *EM* to *EP* isomers, as shown in Table 1, we can expect that the molecular motor DDPY may exceed the barriers in timescales of femtoseconds and arrive at more stable *ZP* or *EP* isomers without staying at the *ZM* or *EM* metastable isomer at room or even lower temperature. Thus, the molecular motor DDPY could complete a full 360${}^{\circ }$ rotation by only two photoisomerization steps (*EP*→*ZP* and *ZP*→*EP*) at room or even lower temperature. The schematic diagram of working cycle for photon-only two-stroke LDMRM DDPY is presented in Figure 1c.

### 3.2. Nonadiabatic Molecular Dynamics Simulations

To verify whether DDPY can work as a photon-only two-stroke LDMRM, nonadiabatic molecular dynamics simulation was systematically carried out. A total of 297 and 325 trajectories starting from the S${}_{1}$ excited state of *EP* and *ZP* isomers at 300 K were firstly studied on the OM2/MRCI level. In addition, 82 of 297 trajectories experienced *EP*→*ZP* photoisomerization, in which 61 trajectories finished the photoisomerization through counterclockwise rotation. Meanwhile, 233 of 325 trajectories underwent *ZP*→*EP* photoisomerization, in which 168 trajectories finished the photoisomerization through counterclockwise rotation. Thus, the unidirectionalities of successful *EP*→*ZP* and *ZP→EP* photoisomerization processes were estimated to be about 74% and 72%, respectively.

**Figure 2:**
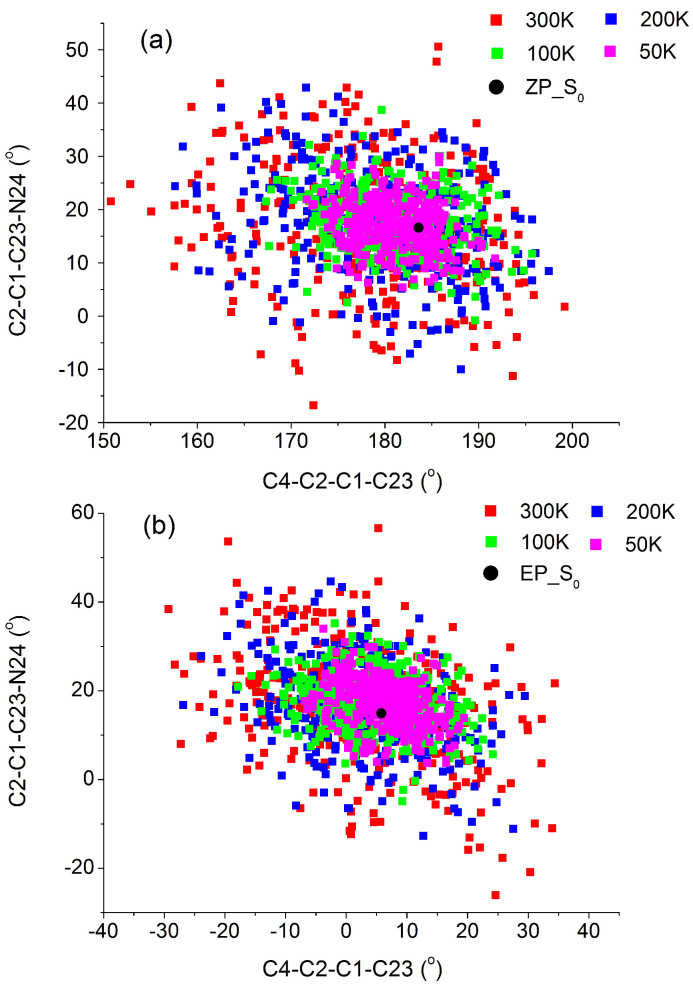
The distribution of C4-C2-C1-C23 and C2-C1-C23-N24 dihedral angles of all sampled initial geometries in Franck–Condon region for (**a**) *ZP*→*EP* photoisomerization and (**b**) *EP*→*ZP* photoisomerization at 300 K, 200 K, 100 K and 50 K. The corresponding points of ground state *ZP* and *EP* isomers are also presented in this figure.

The low unidirectionality may be oweing to the less steric repulsion between the lower half and the upper half of molecular motor DDPY, which results in nearly planar ground state conformations. Low unidirectionality of DDPY at ambient temperature may reduce its application potential as a light-driven molecular rotary motor. How can the unidirectionality of this LDMRM be improved, especially using the physical method? Does temperature influence the nonadiabatic dynamics of molecular motor DDPY? To answer these questions, the ground state dynamics samplings at different temperatures for *EP* and *ZP* isomers were carried out. A total of 328, 341, 291, and 297 geometries for *EP* isomer and 334, 310, 304, and 325 geometries for *ZP* isomer at 50 K, 100 K, 200 K and 300 K, respectively, were randomly selected and chosen by a filtering procedure according to the computed S${}_{0}$-S${}_{1}$ transition probabilities. The distributions of C4-C2-C1-C23 and C2-C1-C23-N24 dihedral angles of all sampled geometries at different temperatures are illustrated in Figure 2. As we can see, distributions of the sampled geometries become closer to the stable *EP* and *ZP* geometries as the temperature decreases. We conjecture that the unidirectionality of this LDMRM may be improved by reducing the temperature. On the basis of trajectory surface-hopping simulation at the semi-empirical OM2/MRCI level, the *EP*→*ZP* and *ZP→EP* nonadiabatic photoisomerization dynamics of DDPY at different temperatures were systematically studied in the following.

#### 3.2.1. The Nonadiabatic Dynamics of *EP*→ *ZP* Photoisomerization

A total of 328, 341, 291, and 297 trajectories starting from the S${}_{1}$ excited state of *EP*-DDPY were performed at the OM2/MRCI level for 1000 fs at 50 K, 100 K, 200 K, and 300 K, respectively. The excited state of S${}_{1}$ corresponds to the single-electron excitation from the HOMO (bonding π orbital) to the LUMO (antibonding π* orbital), with the excitation wavelength at about 377 nm based on the OM2/MRCI level. All trajectories reached the S${}_{0}$ ground state within 1000 fs. In addition, 104, 105, 100, and 82 trajectories underwent *EP*→*ZP* photoisomerization at 50 K, 100 K, 200 K, and 300 K, respectively, which means the quantum yields of *EP*→*ZP* photoisomerization at 50 K, 100 K, 200 K, and 300 K are estimated to be about 32%, 31%, 34%, and 28%, respectively. This indicates that a decrease in temperature has little influence on the quantum yields of *EP*→*ZP* photoisomerization process of DDPY.

**Figure 3:**
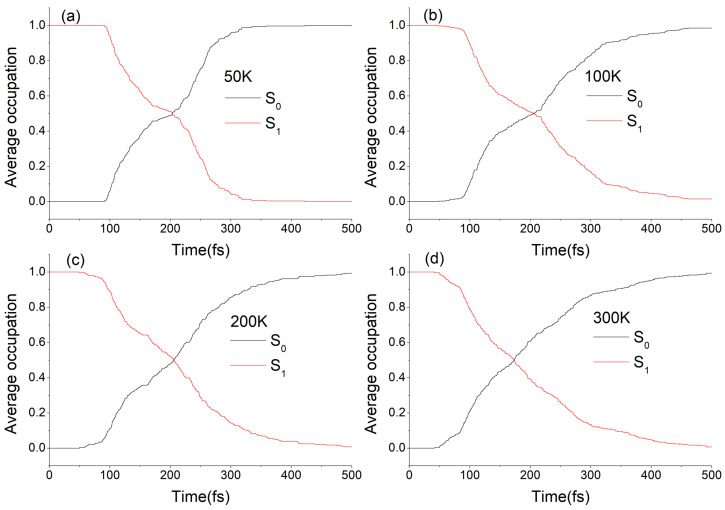
Average occupation of the electronic states S${}_{0}$ and S${}_{1}$ as a function of simulation time in *EP*→*ZP* photoisomerization of DDPY at (**a**) 50 K, (**b**) 100 K, (**c**) 200 K, and (**d**) 300 K, respectively.

The average occupation of electronic states S${}_{0}$ and S${}_{1}$ varying with simulation time at different temperatures are shown in Figure 3. The S${}_{1}$ time-dependent fractional occupation at different temperatures can all be fitted by a bi-exponential function, as shown in Figure S3 (see ESI†), which indicates that the decay modes of molecular motor DDPY are nearly not affected by lowering the temperature. Based on the S${}_{1}$ excited state lifetimes of all 328, 341, 291, and 297 trajectories at 50 K, 100 K, 200 K, and 300 K, average lifetimes of the S${}_{1}$ excited state of the *EP*-DDPY on above temperatures are estimated to be about 192 fs, 210 fs, 206 fs and 191 fs, respectively. The results show that lowering the temperature does not have a significant impact on the decay mode and average S${}_{1}$ lifetime of *EP*→*ZP* photoisomerization process of molecular motor DDPY.

**Figure 4:**
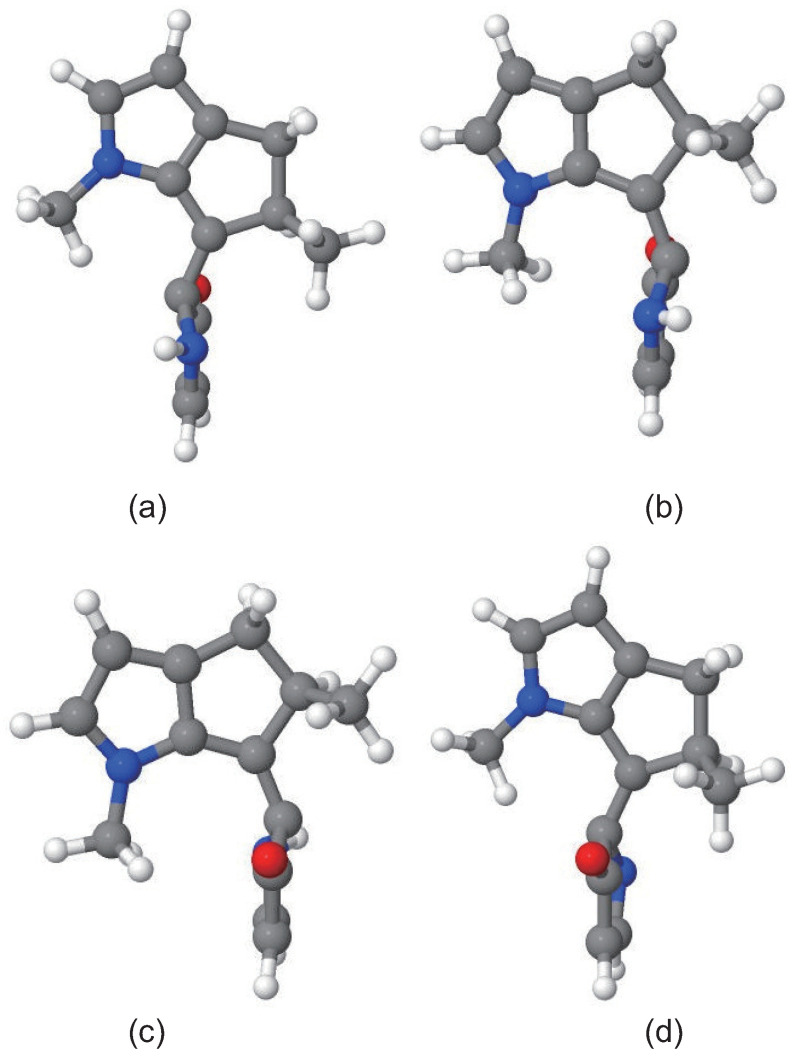
Optimized geometries of four S${}_{1}$/S${}_{0}$ conical intersections (**a**) ECI(1), (**b**) ECI(2), (**c**) ZCI(1), and (**d**) ZCI(2) in the *EP*→*ZP* and *ZP*→*EP* photoisomerization processes calculated with the OM2/MRCI method implemented in the MNDO99 program.

Based on all geometries at the S${}_{1}$/S${}_{0}$ hopping events, four optimized S${}_{1}$/S${}_{0}$ conical intersections (CIs) were obtained at the OM2/MRCI level, as shown in Figure 4, while the corresponding geometrical parameters are summarized in Table S3 (see SI†). According to the characteristic dihedral angle C4-C2-C1-C23 (108.3${}^{\circ }$, 55.7${}^{\circ }$, −56.5${}^{\circ }$ and −109.3${}^{\circ }$, the atomic labels can be seen in Figure 1a), and the four CIs are called *E*CI(1), *E*CI(2), *Z*CI(1), and *Z*CI(2), respectively. As can be seen in Table S3, all CIs involve obvious pyramidalization at the C2 atom site. Similar pyramidalization of the carbon atom at the stator-axle linkage was also observed in other molecular rotary motors [13,25,34,54].

**Figure 5:**
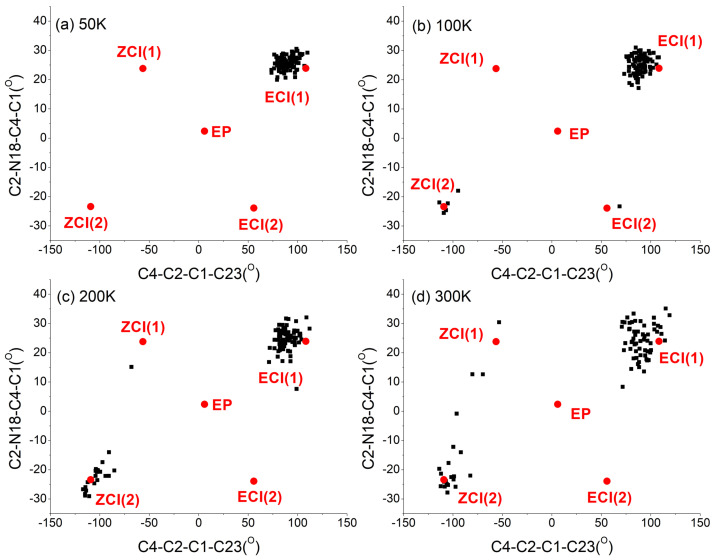
Distribution of the C4-C2-C1-C23 and C2-N18-C4-C1 dihedral angles at the hopping events of successful *EP*→*ZP* photoisomerization trajectories starting from the *EP* structure of DDPY at (**a**) 50 K, (**b**) 100 K, (**c**) 200 K, and (**d**) 300 K, respectively. The corresponding points of ground state *EP* isomer, S${}_{1}$/S${}_{0}$ conical intersections *E*CI(1), *E*CI(2), *Z*CI(1), and *Z*CI(2) are also presented in the figure.

It is helpful for us to understand the decay mechanism through distribution of geometrical parameters at hopping events. For the trajectories experienced *EP*→*ZP* photoisomerization at 50 K, 100 K, 200 K, and 300 K, the distributions of C4-C2-C1-C23 and C2-N18-C4-C1 dihedral angles at the S${}_{1}$ → S${}_{0}$ hopping events are illustrated in Figure 5. For all trajectories at 50 K, 100 K, 200 K, and 300 K, the distributions of C4-C2-C1-C23 and C2-N18-C4-C1 dihedral angles at the S${}_{1}$→S${}_{0}$ hopping events are also illustrated in Figure S5 (see SI†). The corresponding points of the ground state *EP*-isomer, conical intersections *E*CI(1), *E*CI(2), *Z*CI(1), and *Z*CI(2) are also presented in Figure 5 and Figure S5.

Taking 300 K as an example, as shown in Figure 5d, most of the trajectories rotate counterclockwise and some trajectories rotate clockwise. The trajectories of counterclockwise rotation experienced *EP*→*ZP* photoisomerization were accessed through hops close to the *E*CI(1). The trajectories of clockwise rotation experienced *EP*→*ZP* photoisomerization were accessed through hops close to the *Z*CI(1) and *Z*CI(2). Some hops close to the initial *EP* structure were also observed, as shown in Figure S5d, but all corresponding trajectories returned to the reactant.

As shown in Figure 5, as the temperature decreases, the proportion of trajectories that rotates clockwise became smaller and smaller. For example, at 50 K, all trajectories that experienced *EP*→*ZP* photoisomerization underwent counterclockwise rotation. The statistical unidirectionalities of the successful *EP*→*ZP* photoisomerization process are 74%, 77%, 95% and 100% at 300K, 200 K, 100 K, and 50 K, respectively. Thus, lowering the temperature can significantly increase the unidirectionality of the *EP*→*ZP* photoisomerization process of molecular motor DDPY.

**Figure 6:**
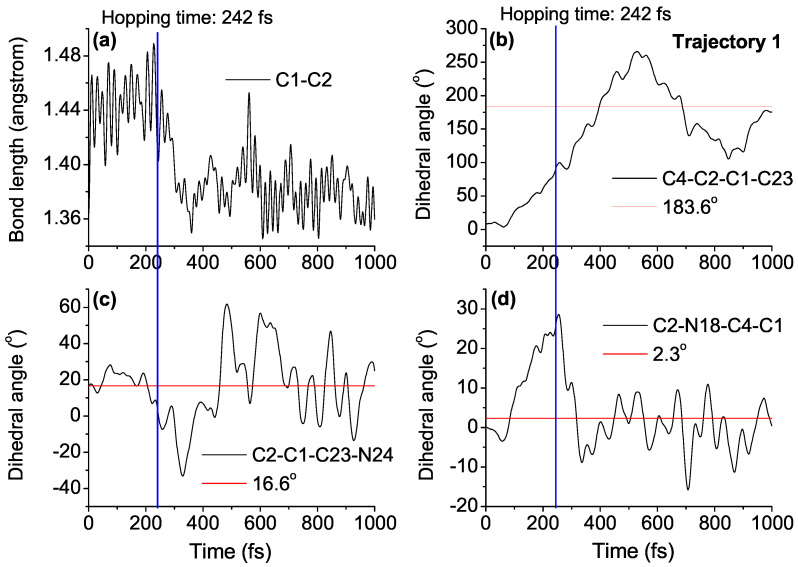
Time dependence of (**a**) central bond length C1-C2; (**b**) central dihedral angle C4-C2-C1-C23; (**c**) side dihedral angle C2-C1-C23-N24; and (**d**) pyramid dihedral angle C2-N18-C4-C1 in a representative trajectory (called trajectory 1) of *EP*→*ZP* photoisomerization process. The S${}_{1}$→S${}_{0}$ hopping time (blue line) and corresponding geometrical parameters of reaction product *ZP* isomer (red lines) are also shown in the figure.

In order to understand the *EP*→*ZP* photoisomerization mechanism of DDPY in detail, time-dependent evolutions of central bond length C1-C2, central dihedral angle C4-C2-C1-C23, side dihedral angle C2-C1-C23-N24, and pyramid dihedral angle C2-N18-C4-C1 in five typical trajectories (called trajectories 1–5, respectively) at 50 K are presented in Figure 6 and Figures S7–S10 (see ESI†). The corresponding geometrical parameters of reaction product *ZP* isomer and S${}_{1}$→S${}_{0}$ hopping time are also shown in the figures.

Take trajectory 1 as an example, as shown in Figure 6, after the excitation from S${}_{0}$ to S${}_{1}$, the central C1=C2 double bond is weakened, increasing from its optimized ground state value of 1.38 Å to about 1.46 Å, varying around 1.44 Å until the nonadiabatic decay at 242 fs, then returning to about 1.38 Å. That is, the excitation from the bonding π orbital of the central C=C bond to the antibonding π* orbital reduces its double bond character obviously. The dihedral angle C4-C2-C1-C23 increased gradually from 9.1${}^{\circ }$ to about 95.1${}^{\circ }$ around 242 fs, after the de-excitation, it increased continually to its optimized ground state value of 183.6${}^{\circ }$ in the *ZP* structure at about 400 fs. The dihedral angle C2-N18-C4-C1, characterizing the pyramidalization at the C2 atom, increased to 24.5${}^{\circ }$ when nonadiabatic decay occurred at 242 fs, then decreasing dramatically to 2.3${}^{\circ }$ at about 310 fs, and varying around 2.3${}^{\circ }$ until the end of simulation. Both optimized geometries of conical intersection presented in Figure 4 and the time dependence of geometrical parameters shown in Figure 6 verify that, after the S${}_{0}$→S${}_{1}$ excitation, the dynamical process of nonadiabatic decay is followed by twisting about the central C=C double bond and the pyramidalization of the C atom at the stator–axle linkage.

Side dihedral angle C2-C1-C23-N24 is the key geometrical parameter to distinguish *ZP* and *ZM* isomers of DDPY, as can be seen in Table S1 (see ESI†). As shown in Figure 6c, the dihedral angle C2-C1-C23-N24 vibrated around 14.9${}^{\circ }$ (optimized value in *EP* geometry) until the nonadiabatic decay at 250 fs, then decreasing dramatically to –1.8${}^{\circ }$ (optimized value in *ZM* geometry) at about 300 fs, i.e., molecular motor arrived at the *ZM* geometry. After staying around the *ZM* geometry for less than 200 fs, the dihedral angle C2-C1-C23-N24 increased continually to 16.6${}^{\circ }$ (optimized value in *ZP* geometry) at about 460 fs, and vibrated around this value until the end of simulation. Time dependence of dihedral angle C2-C1-C23-N24 shown in Figure 6c verifies that, after the S${}_{0}$→S${}_{1}$ excitation of *EP* isomer, molecular motor DDPY arrives at the *ZM* isomer firstly, then reaching the *ZP* isomer in a very short time. For example, the *EP*→*ZP* photoisomerization process of DDPY can be realized at 50 K or even lower temperatures, which confirms our expectation in the beginning.

#### 3.2.2. The Nonadiabatic Dynamics of *ZP→EP* Photoisomerization

With the same method as the above *EP*→*ZP* photoisomerization nonadiabatic dynamics simulation, nonadiabatic dynamics of *ZP→EP* photoisomerization was systemically investigated. At 50 K, 100 K, 200 K, and 300 K, molecular dynamics simulations of 334, 310, 304, and 325 trajectories starting from the S${}_{1}$ excited state (with the excitation wavelength at about 353 nm) were performed at the OM2/MRCI level for 1000 fs, respectively. All trajectories decayed to the ground state before the end of simulation. A total of 288, 240, 232, and 233 trajectories underwent *ZP→EP* photoisomerization at 50 K, 100 K, 200 K, and 300 K, respectively, which means the quantum yields of *ZP→EP* photoisomerization at the above corresponding temperatures are estimated to be about 86%, 77%, 76%, and 72%, respectively. This indicates that the effect of temperature on the quantum yields of *ZP→EP* photoisomerization process of molecular motor DDPY is not significant.

**Figure 7:**
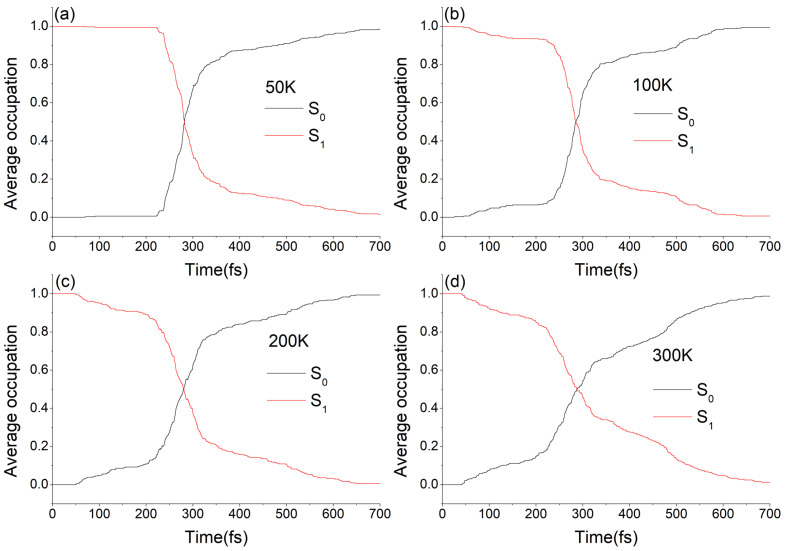
Average occupation of the electronic states S${}_{0}$ and S${}_{1}$ as a function of simulation time in *ZP→EP* photoisomerization process of DDPY at (**a**) 50 K, (**b**) 100 K, (**c**) 200 K, and (**d**) 300 K, respectively.

The average occupation of electronic states S${}_{0}$ and S${}_{1}$ varying with simulation time at different temperatures are shown in Figure 7. As we can see, the S${}_{1}$ population decay at different temperatures are obviously not exponential. Taking a numerical derivative on the occupation of S${}_{0}$ state over time at different temperatures, as shown in Figure S4 (see SI†), the decay mode of the S${}_{1}$ excited state are all found to be periodic. Taking 300 K as an example, as is shown in Figure 7d and Figure S4d, four major hopping event maxima arose around 50 fs, 270 fs, 490 fs and 690 fs, respectively. This indicates that the motion of the molecular motor on the S${}_{1}$ excited state towards the conical intersection is regulated by a periodic structural change. The periodic intervals of hopping event maxima in Figure S4d are roughly in 200–220 fs range, close to a ground state normal mode of *ZP*-DDPY (148 cm${}^{-1}$, the fifth normal mode, corresponding vibrational duration is 225 fs) involving a swing of phenmethyl ring around the central C=C double bond. Similar periodic decay modes have also been observed in *Z-E* photoisomerization of some azobenzene-based molecules [40,42,43]. Based on the S${}_{1}$ excited state lifetimes of all 334, 310, 303, and 324 trajectories at 50 K, 100 K, 200 K, and 300 K in our calculations, average lifetime of the S${}_{1}$ excited state of the *ZP*-DDPY is estimated to be about 316 fs, 310 fs, 300 fs, and 322 fs, respectively. The results show that lowering the temperature does not have a significant impact on the decay mode and average S${}_{1}$ lifetime of *ZP→EP* photoisomerization dynamics process of molecular motor DDPY.

**Figure 8:**
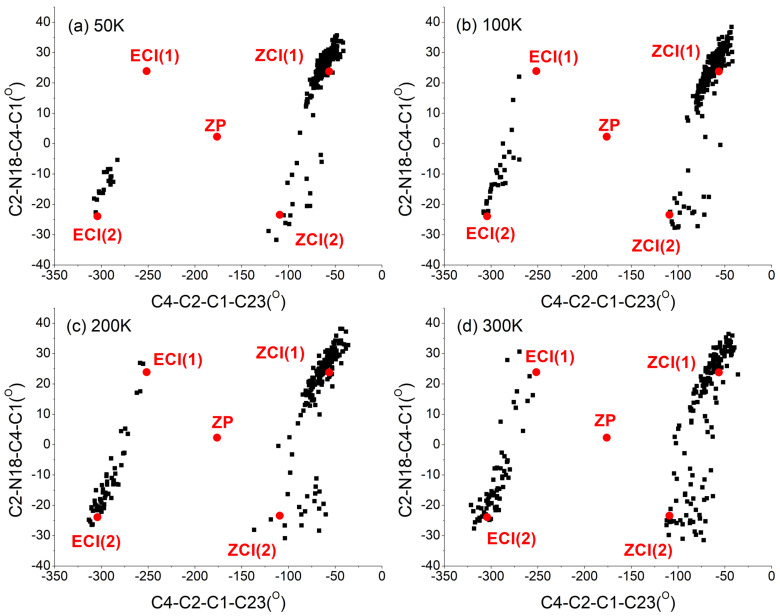
Distribution of the C4-C2-C1-C23 and C2-N18-C4-C1 dihedral angles at the hopping events of successful *ZP→EP* photoisomerization trajectories starting from the *ZP* structure of DDPY at (**a**) 50 K; (**b**) 100 K; (**c**) 200 K; and (**d**) 300 K, respectively. The ground state *ZP* isomer, *E*CI(1), *E*CI(2), *Z*CI(1), and *Z*CI(2) are also presented in this figure at different temperatures.

Based on all geometries at the S${}_{1}$/S${}_{0}$ hopping events, four optimized S${}_{1}$/S${}_{0}$ conical intersections were obtained at the OM2/MRCI level, which are the same as those obtained in the above *EP*→*ZP* photoisomerization process. For the trajectories experienced *ZP→EP* photoisomerization at 50 K, 100 K, 200 K, and 300 K, the distributions of C4-C2-C1-C23 and C2-N18-C4-C1 dihedral angles at the S${}_{1}$→S${}_{0}$ hopping events are illustrated in Figure 8. The distributions of C4-C2-C1-C23 and C2-N18-C4-C1 dihedral angles at the S${}_{1}$→S${}_{0}$ hopping events for all trajectories at 50 K, 100 K, 200 K, and 300 K are presented in Figure S6 (see SI†). The corresponding points of ground state *ZP*-isomer, conical intersections *E*CI(1), *E*CI(2), *Z*CI(1), and *Z*CI(2) are also displayed in Figure 8 and Figure S6.

Taking 300 K as an example, as shown in Figure 8d, most of the trajectories rotated counterclockwise and some of the trajectories rotated clockwise. The trajectories of counterclockwise rotation experienced *ZP→EP* photoisomerization were accessed through hops close to the *Z*CI(1) and *Z*CI(2). The trajectories of clockwise rotation experienced *ZP→EP* photoisomerization were accessed through hops close to the *E*CI(1) and *E*CI(2). Although a few hops close to the initial *ZP* structure were also observed, as shown in Figure S6d, all the corresponding trajectories returned to the reactant *ZP* isomer and did not experience the *ZP→EP* photoisomerization. As shown in Figure 8, as the temperature decreases, the proportion of clockwise rotation trajectories became smaller and smaller. The statistical unidirectionalities of the trajectories which experienced *ZP→EP* photoisomerization are 72%, 76%, 88% and 94% at 300 K, 200 K, 100 K, and 50 K, respectively. The same as *ZP→EP* photoisomerization of molecular motor DDPY, the unidirectionality of *ZP→EP* photoisomerization process can also be significantly improved through lowering the temperature.

**Figure 9:**
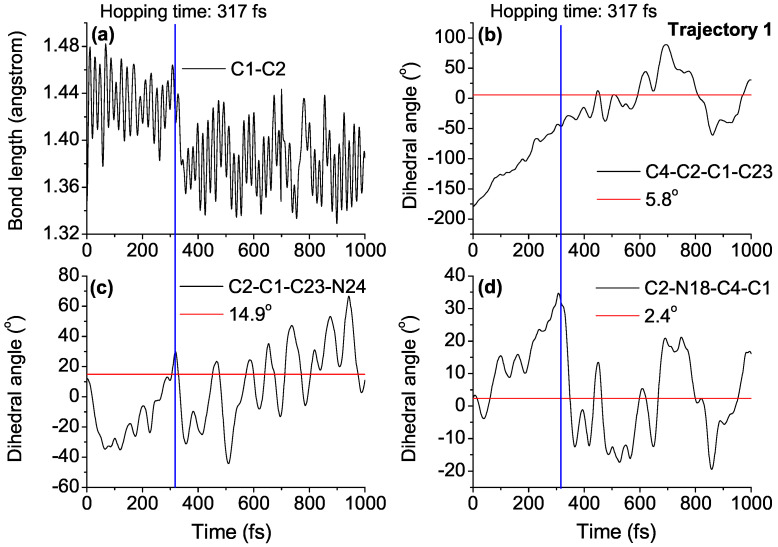
Time dependence of (**a**) central bond length C1-C2; (**b**) central dihedral angle C4-C2-C1-C23; (**c**) side dihedral angle C2-C1-C23-N24; and (**d**) pyramid dihedral angle C2-N18-C4-C1 in a representative trajectory (named as trajectory 1) of *ZP*→*EP* photoisomerization process. The S${}_{1}$→S${}_{0}$ hopping time (blue line) and correponding geometrical parameters of reaction product *EP* isomer (red lines) are also shown in the figure.

In order to understand the *ZP→EP* photoisomerization mechanism of DDPY in detail, time-dependent evolutions of central bond length C1=C2, central dihedral angle C4-C2-C1-C23, side dihedral angle C2-C1-C23-N24, and pyramid dihedral angle C2-N18-C4-C1 in five typical trajectories (named as trajectory 1–5, respectively) at 50 K are presented in Figure 9 and Figures S11–S14 (see ESI†). The corresponding geometrical parameters of reaction product *EP* isomer and S${}_{1}$→S${}_{0}$ hopping time are also shown in the figures.

Take trajectory 1 as an example, as shown in Figure 9, after the excitation from S${}_{0}$ to S${}_{1}$, the central C1=C2 double bond is weakened, increasing from its optimized ground state value of 1.37 Å to about 1.48 Å, varying around 1.44 Å until the nonadiabatic decay at 317 fs, then returning to about 1.37 Å. The dihedral angle C4-C2-C1-C23 increased gradually from −179.1${}^{\circ }$ to about −46.7${}^{\circ }$ around 317 fs; after the de-excitation, it increased continually to its optimized ground state value of 5.8${}^{\circ }$ in the *EP* structure at about 450 fs. The dihedral angle C2-N18-C4-C1, characterizing the pyramidalization at the C2 atom, increased to 29.7${}^{\circ }$ when nonadiabatic decay occurred at 317 fs, then decreasing dramatically to 2.4${}^{\circ }$ around 350 fs, and varying around 2.4${}^{\circ }$ until the end of simulation. The time dependence of geometrical parameters shown in Figure 9, together with optimized geometries of conical intersections presented in Figure 4, verify that the dynamical process of nonadiabatic decay is followed by twisting about the central C=C double bond and the pyramidalization of the C atom at the stator–axle linkage.

Side dihedral angle C2-C1-C23-N24 is the key geometrical parameter to distinguish *EP* and *EM* isomers of DDPY, as can be seen in Table S1 (see ESI†). As shown in Figure 9, the dihedral angle C2-C1-C23-N24 decreased dramatically from 16.6${}^{\circ }$ (optimized value in *ZP* geometry) to −35${}^{\circ }$ within 75 fs and then increased gradually to 20${}^{\circ }$ at 317 fs. After the nonadiabatic decay at about 317 fs, the dihedral angle C2-C1-C23-N24 decreased dramatically to −6.1${}^{\circ }$ (optimized value in *EM* geometry) at about 340 fs, i.e., molecular motor arrived at the *EM* geometry. After staying around the *EM* geometry for about 200 fs, the dihedral angle C2-C1-C23-N24 increased to 14.9${}^{\circ }$ (optimized value in *EP* geometry) at about 560 fs and then vibrated around this value until the end of simulation. It suggests that the *ZP→EP* photoisomerization process of DDPY can also be realized at 50 K or even lower temperatures, which also confirms our expectation in the beginning.

Before the conclusions, average lifetime of the S${}_{1}$ excited state, quantum yield, and unidirectionality at different temperatures for the *EP*→*ZP* and *ZP→EP* photoisomerization of molecular motor DDPY are summarized in Table 2 for comparison. As we can see, as the temperature decreases, average lifetimes of the S${}_{1}$ excited state and quantum yield of both *EP*→*ZP* and *ZP→EP* photoisomerization are almost unaffected, while the unidirectionalities are significantly increased.

## 4. Conclusions

Based on electronic structure calculation at the B3LYP/6-31G(d), CAM-B3LYP/6-31G(d), B3LYP-D3/6-31+G(d), and OM2/MRCI level, together with nonadiabatic molecular dynamics simulation at the OM2/MRCI level, a two-stroke light-driven molecular rotary motor, 2-(1,5-dimethyl-4,5-dihydrocyclopenta[b]pyrrol-6(1H)-ylidene)-1,2-dihydro-3H-pyrrol-3-one (DDPY) is proposed, which is capable of completing a unidirectional 360${}^{\circ }$ rotation by only two photoisomerization (*EP→ZP* and *ZP→EP*) steps. The nonadiabatic dynamics of *EP*→*ZP* and *ZP→EP* photoisomerization of DDPY are investigated by trajectory surface-hopping molecular dynamics at the OM2/MRCI level. Both photoisomerization processes are on an ultrafast timescale (ca. 200–300 fs). The decay mode of *EP*→*ZP* photoisomerization is approximately bi-exponential, while the decay mode of *ZP→EP* photoisomerization was found to be periodic. For *EP* and *ZP* isomer of DDPY, after the S${}_{0}$→S${}_{1}$ excitation, the dynamical processes of nonadiabatic decay are both followed by twisting about the central C=C double bond and the pyramidalization of the C atom at the stator–axle linkage.

The effect of temperature on the nonadiabatic dynamics of *EP*→*ZP* and *ZP→EP* photoisomerization of DDPY has been systematically investigated. Based on a large number of trajectories starting from the S${}_{1}$ excited state of *EP* and *ZP* isomer at 50 K, 100 K, 200 K, and 300 K, we found that average lifetimes of the S${}_{1}$ excited state and quantum yields for both *EP*→*ZP* and *ZP→EP* photoisomerization are almost temperature-independent, while the corresponding unidirectionality of rotation is significantly increased as the temperature decreases. Our present computational results not only proposed a new class of two-stroke photon-only light-driven molecular rotary motor, but also supplied a physical way to increase the unidirectionality of molecular motor, which may stimulate further research for the development of more efficient light-driven molecular rotary motors.

## Figures and Tables

**Table 1:** Energy barriers from *EM* to *EP* isomers and from *ZM* to *ZP* isomers in the ground state, obtained from the OM2/MRCI, B3LYP/6-31G(d), CAM-B3LYP/6-31G(d), and B3LYP-D3/6-31+G(d) methods. The energy unit is kcal/mol.

	OM2/MRCI	B3LYP/6-31G(d)	CAM-B3LYP/6-31G(d)	B3LYP-D3/6-31+G(d)
*EM→EP*	0.17	0.20	0.05	0.31
*ZM→ZP*	0.09	0.60	0.56	0.47

**Table 2:** The average S${}_{1}$ lifetime, quantum yield, and unidirectionality for *EP*→*ZP* and *ZP→EP* photoisomerization nonadiabatic dynamics simulation at 300 K, 200 K, 100 K, and 50 K, respectively.

	Temperature	Average S${}_{1}$ Lifetime	Quantum Yield	Unidirectionality
*EP*→*ZP*	300 K	191 fs	28%	74%
	200 K	206 fs	34%	77%
	100 K	210 fs	31%	95%
	50 K	192 fs	32%	100%
*ZP→EP*	300 K	322 fs	72%	72%
	200 K	300 fs	76%	76%
	100 K	310 fs	77%	88%
	50 K	316 fs	86%	94%

## Data Availability

Not applicable.

## Supplementary Materials

The supporting information can be downloaded at: https://www.mdpi.com/article/10.3390/ijms23179694/s1.
