# Supplementary material for: Effect of Temperature on Photoisomerization Dynamics of a Newly Designed Two-Stroke Light-Driven Molecular Rotary Motor
Source: Int J Mol Sci. 2022 Aug 26;23(17):9694. doi: 10.3390/ijms23179694 (PMC9456002; doi:10.3390/ijms23179694)
Supplement: Supplementary file 1 [file ijms-23-09694-s001.zip › ijms-1869364-supplementary.pdf]

Supporting Information (SI) For:

Effect of Temperature on Photoisomerization Dynamics of a Newly Designed Two-Stroke Light-Driven Molecular Rotary Motor

Jianzheng Ma[1], Di Zhao[1], Chenwei Jiang[1,*], Zhenggang Lan[2,*], and Fuli Li[1]

[1]Ministry of Education Key Laboratory for Nonequilibrium Synthesis and Modulation of Condensed Matter, Shaanxi Province Key Laboratory of Quantum Information and Quantum Optoelectronic Devices, School of Physics, Xi'an Jiaotong University, Xi'an 710049, China

[2]Guangdong Provincial Key Laboratory of Chemical Pollution and Environmental Safety & MOE Key Laboratory of Environmental Theoretical Chemistry, SCNU Environmental Research Institute, School of Environment, South China Normal University, Guangzhou 510006, China

**Corresponding authors. Email: jiangcw@xjtu.edu.cn (C.J.); zhenggang.lan@m.scnu.edu.cn (Z. L.).*

Table of contents

1. Optimized ground state and transition state geometries of two-stroke LDMRM DDPY..S2

2. Optimized geometries of conical intersection in the photoisomerization processes of two-stroke LDMRM DDPY..S5

3. The decay mode of EP→ZP and ZP→EP photoisomerization processes of LDMRM DDPY...S6

4. The distribution of geometries at the hopping events of all trajectories for EP→ZP and ZP→EP photoisomerization processes of LDMRM DDPY…………………………………………………………..................S8

5. Time-dependent evolution of geometrical parameters in several typical trajectories of two-stroke LDMRM DDPY at 50 K..............................S10

6. Cartesian coordinates for several structures of two-stroke LDMRM DDPY optimized with the OM2/MRCI method...................................S19

7. Cartesian coordinates for several structures of two-stroke LDMRM DDPY optimized with the B3LYP/6-31G(d) method...........................S27

8. Cartesian coordinates for several structures of two-stroke LDMRM DDPY optimized with the CAM-B3LYP/6-31G(d) method.................S32

9. Cartesian coordinates for several structures of two-stroke LDMRM DDPY optimized with the B3LYP-D3/6-31G+(d) method..................S37

10. References.................………………..S42

1. Optimized ground state and transition state geometries of two-stroke LDMRM DDPY

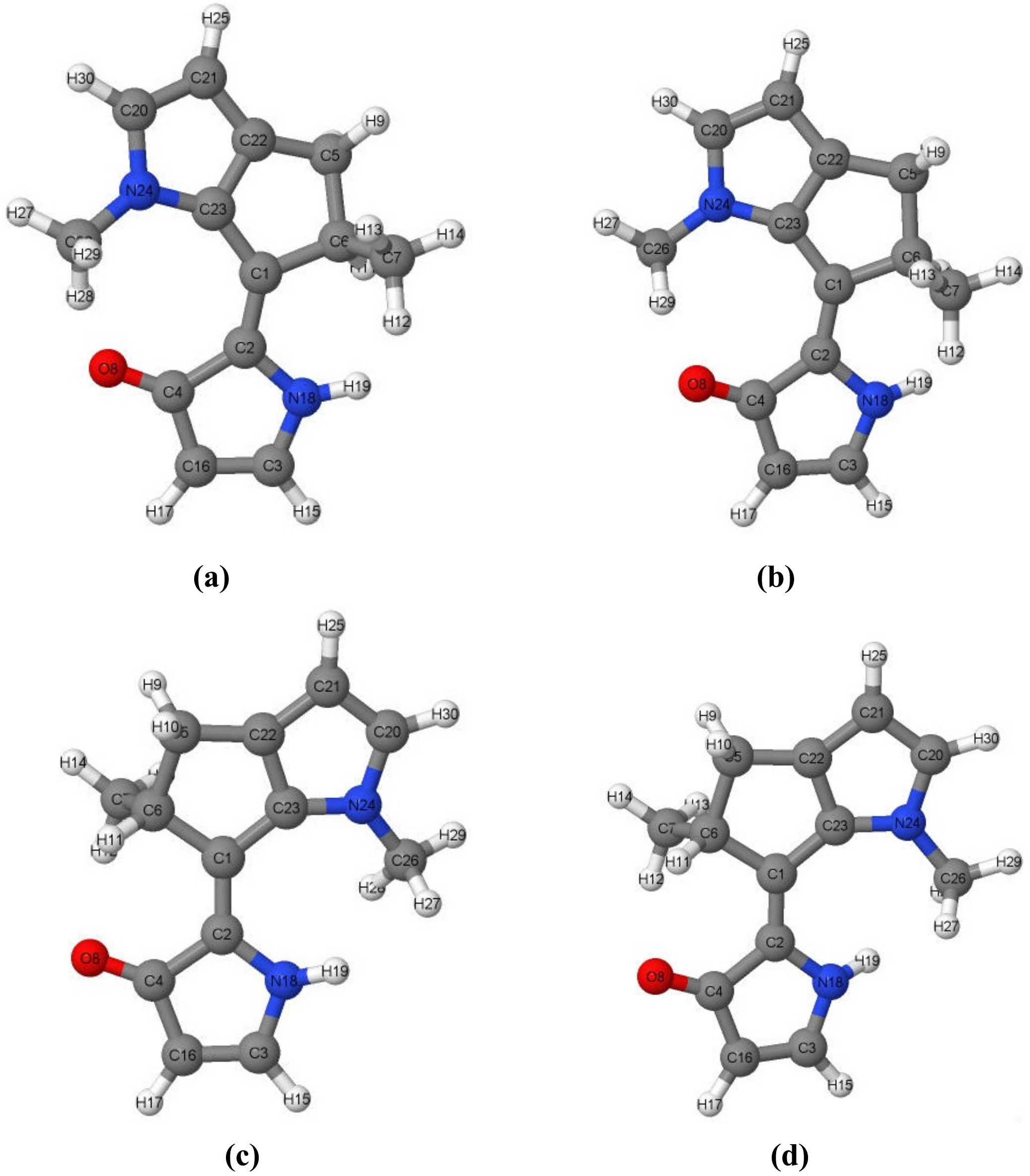

Figure S1. Optimized ground state geometries of (a) *EP*, (b) *EM*, (c) *ZP* and (d) *ZM* isomers calculated with the OM2/MRCI method implemented in MNDO99[S1] program. All atoms are labelled.

Table S1. Optimized geometrical parameters of ground state two-stroke LDMRM DDPY, obtained from different methods. The OM2/MRCI is implemented in MNDO99[S1] program; while B3LYP/6-311G(d), CAM-B3LYP/6-31G(d) and B3LYP-D3/6-31+G(d) are all implemented in Gaussian 09[S2] program. The lengths are in angstroms; the dihedral angles and bond angles are in degrees.

		B3LYP/6-31G(d)	CAM-B3LYP/6-31G(d)	B3LYP-D3/6-31+G(d)	OM2/MRCI
EP	C1-C2	1.37	1.36	1.37	1.38
	C4-C2-C1	132.5	132.2	131.8	134.0
	C2-C1-C6	120.6	120.5	121.1	121.6
	C4-C2-C1-C6	-163.9	-164.8	-161.7	-168.6
	C4-C2-C1-C23	12.3	11.6	14.7	5.8
	C2-N18-C4-C1	4.1	3.9	4.5	2.4
	C2-C1-C23-N24	19.5	19.8	20.7	14.9
EM	C1-C2	1.37	1.36	1.37	1.37
	C4-C2-C1	134.1	134.2	131.7	133.3
	C2-C1-C6	119.3	118.9	121.3	121.0
	C4-C2-C1-C6	159.0	161.5	154.6	163.9
	C4-C2-C1-C23	-21.3	-18.6	-24.9	-20.6
	C2-N18-C4-C1	-3.2	-2.8	-4.2	-2.7
	C2-C1-C23-N24	-2.8	-1.7	-9.9	-6.1
ZP	C1-C2	1.36	1.35	1.36	1.37
	C4-C2-C1	127.5	127.0	127.5	126.4
	C2-C1-C6	123.0	123.3	123.2	123.7
	C4-C2-C1-C6	3.8	3.5	4.9	7.5
	C4-C2-C1-C23	179.8	179.8	181.1	183.6
	C2-N18-C4-C1	-0.5	-0.4	-0.3	2.3
	C2-C1-C23-N24	16.6	16.4	17.7	16.6
ZM	C1-C2	1.36	1.35	1.36	1.37
	C4-C2-C1	126.9	126.5	126.9	125.5
	C2-C1-C6	121.6	122.0	121.6	122.3
	C4-C2-C1-C6	-11.9	-11.0	-13.0	-14.2
	C4-C2-C1-C23	167.9	169.0	167.5	166.0
	C2-N18-C4-C1	-3.1	-3.0	-3.0	-4.0
	C2-C1-C23-N24	2.5	2.9	1.0	-1.8

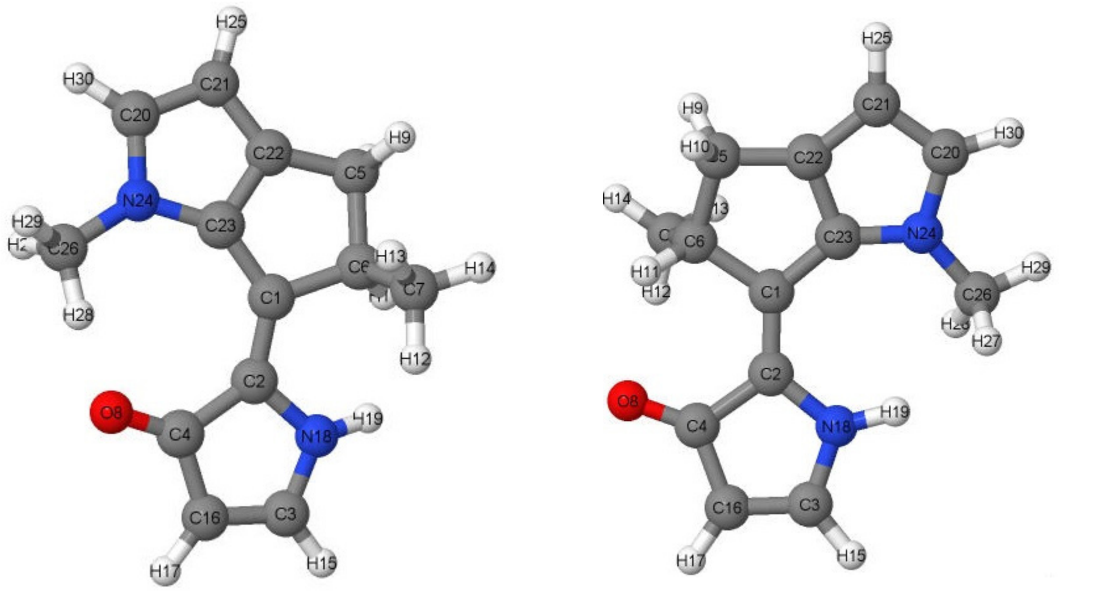

(a) **(b)**

Figure S2. Optimized transition state geometries of (a) *EM-EP-TS* and (b) *ZM-ZP-TS* for ground state DDPY calculated with the OM2/MRCI method implemented in MNDO99[S1] program. All atoms are labelled.

Table S2. Optimized transition state geometrical parameters of ground state two-stroke LDMRM DDPY, obtained from different methods. The OM2/MRCI method is implemented in MNDO99[S1] program; while B3LYP/6-311G(d), CAM-B3LYP /6-31G(d) and B3LYP-D3/6-31+G(d) methods are all implemented in Gaussian 09[S2] program. The lengths are in angstroms; the dihedral angles and bond angles are in degrees.

		B3LYP/6-31G(d)	CAM-B3LYP/6-31G(d)	B3LYP-D3/6-31+G(d)	OM2/MRCI
EM-EP-TS	C1-C2	1.37	1.36	1.37	1.38
	C4-C2-C1	135.4	134.2	133.8	134.7
	C2-C1-C6	118.0	118.6	118.6	120.1
	C4-C2-C1-C6	170.8	165.6	167.7	171.0
	C4-C2-C1-C23	-10.0	-14.9	-12.3	-14.2
	C2-N18-C4-C1	-1.1	-2.0	-1.8	-1.6
	C2-C1-C23-N24	6.2	0.7	2.7	2.3
ZM-ZP-TS	C1-C2	1.36	1.35	1.36	1.37
	C4-C2-C1	126.8	126.5	126.9	126.2
	C2-C1-C6	121.9	122.2	121.9	122.6
	C4-C2-C1-C6	-2.9	-2.7	-2.5	-1.7
	C4-C2-C1-C23	174.7	175.2	175.4	174.1
	C2-N18-C4-C1	-1.8	-1.7	-1.9	-0.8
	C2-C1-C23-N24	11.5	11.4	11.8	13.0

2. Optimized geometries of conical intersection in the photoisomerization processes of two-stroke LDMRM DDPY

Table S3. Optimized geometrical parameters of four S_1/S_0 conical intersections *ECI(1)*, *ECI(2)*, *ZCI(1)* and *ZCI(2)* in the photoisomerization process calculated with the OM2/MRCI method implemented in MNDO99[S1] program. The lengths are in angstroms; the dihedral angles and bond angles are in degrees.

		OM2/MRCI
ECI(1)	C1-C2	1.42
	C4-C2-C1	113.8
	C2-C1-C6	130.2
	C4-C2-C1-C6	-55.5
	C4-C2-C1-C23	108.3
	C2-N18-C4-C1	23.9
	C2-C1-C23-N24	4.6
ECI(2)	C1-C2	1.41
	C4-C2-C1	114.3
	C2-C1-C6	123.9
	C4-C2-C1-C6	-110.8
	C4-C2-C1-C23	55.7
	C2-N18-C4-C1	-23.9
	C2-C1-C23-N24	2.7
ZCI(1)	C1-C2	1.42
	C4-C2-C1	114.6
	C2-C1-C6	124.0
	C4-C2-C1-C6	110.3
	C4-C2-C1-C23	-56.5
	C2-N18-C4-C1	23.8
	C2-C1-C23-N24	-3.1
ZCI(2)	C1-C2	1.42
	C4-C2-C1	115.2
	C2-C1-C6	131.7
	C4-C2-C1-C6	57.6
	C4-C2-C1-C23	-109.3
	C2-N18-C4-C1	-23.4
	C2-C1-C23-N24	-3.7

3. The decay mode of EP→ZP and ZP→EP photoisomerization processes of LDMRM DDPY

As we can see in Figure S3, the natural logarithm of S_1 time-dependent fractional occupation at 50 K, 100 K, 200 K and 300 K can be fitted by two straight lines. *I.e.*, the decay modes of molecular motor DDPY for the EP→ZP photoisomerization process are bi-exponential at different temperature.

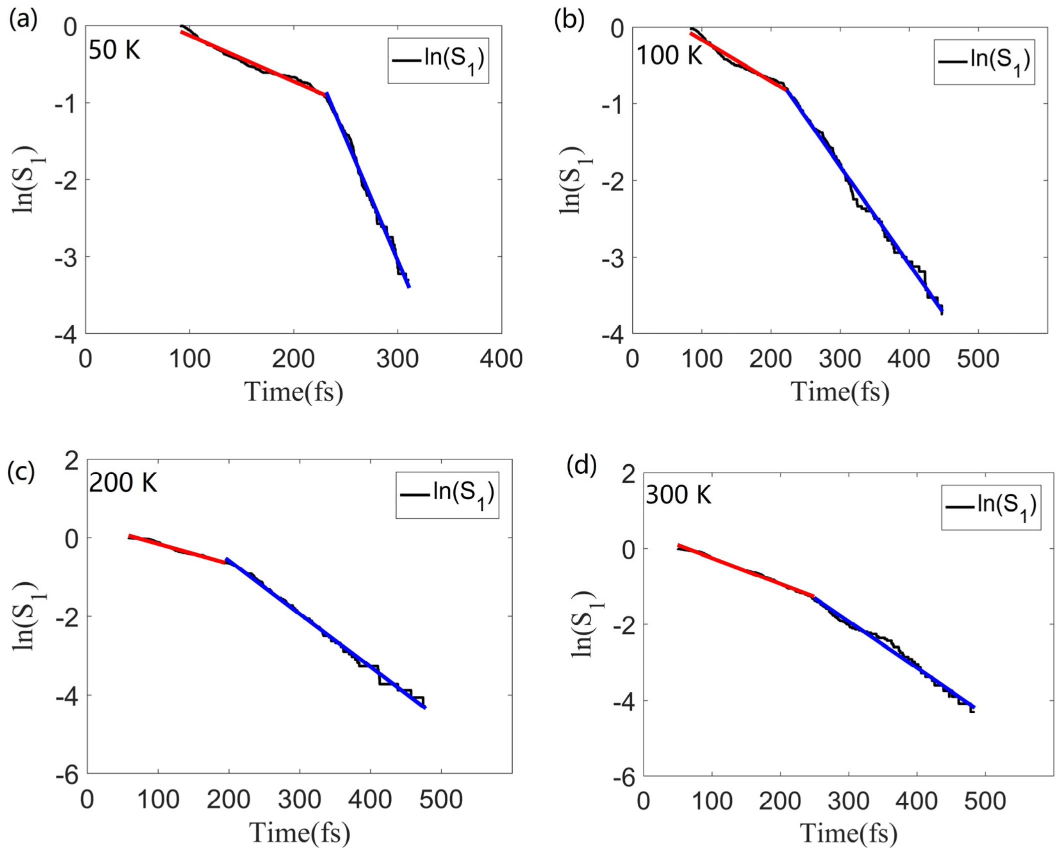

Figure S3. The average occupation of the S_1 excited state over time in the *EP→ZP* photoisomerization process of molecular motor DDPY fitted by bi-exponential functions at (a) 50 K, (b) 100 K, (c) 200 K and (d) 300 K, respectively.

After taking a numerical derivative on the occupation of S_0 state over time at 50 K, 100 K, 200 K and 300 K, respectively, as can be seen in Figure S4, some of the peaks decrease or even disappear as the temperature decreasing. So the decay mode of molecular motor DDPY for the *ZP→EP* photoisomerization process are all periodic at different temperature.

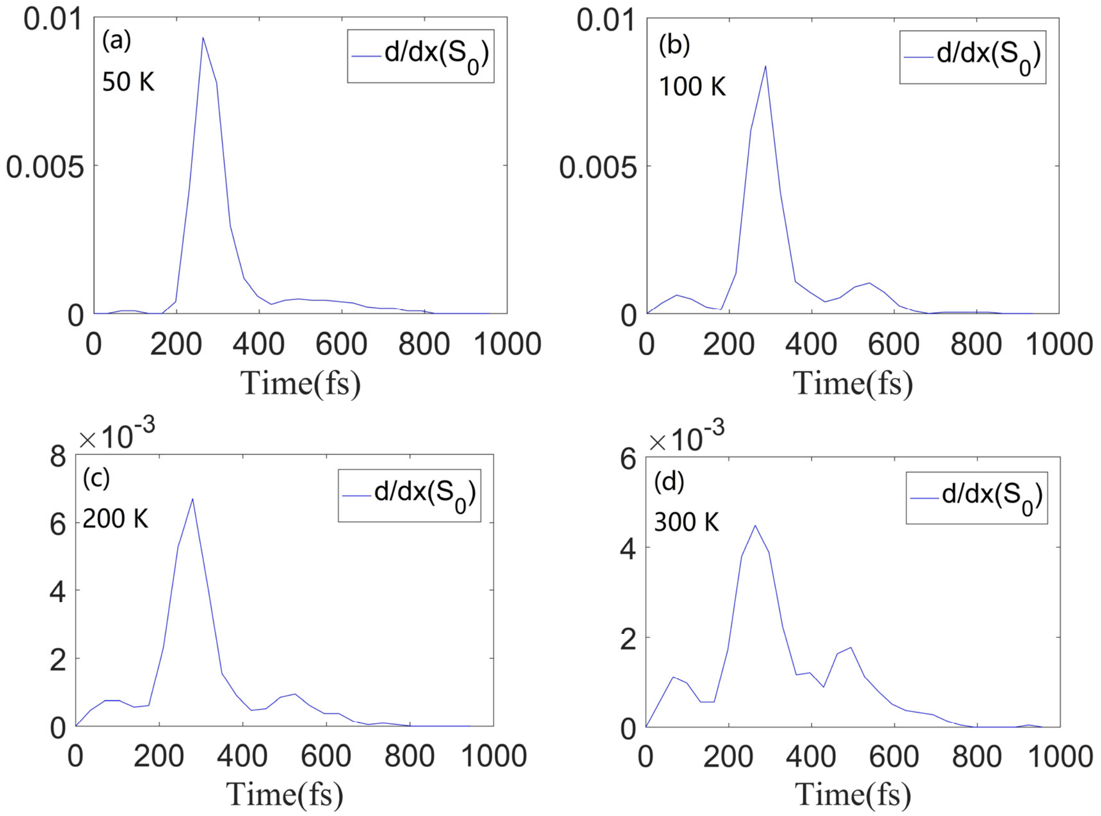

Figure S4. The first derivative of the S_0 population over time in the *ZP→EP* photoisomerization process of molecular motor DDPY at (a) 50 K, (b) 100 K, (c) 200 K and (d) 300 K, respectively.

4. The distribution of geometries at the hopping events of all trajectories for EP→ZP and ZP→EP photoisomerization processes of LDMRM DDPY

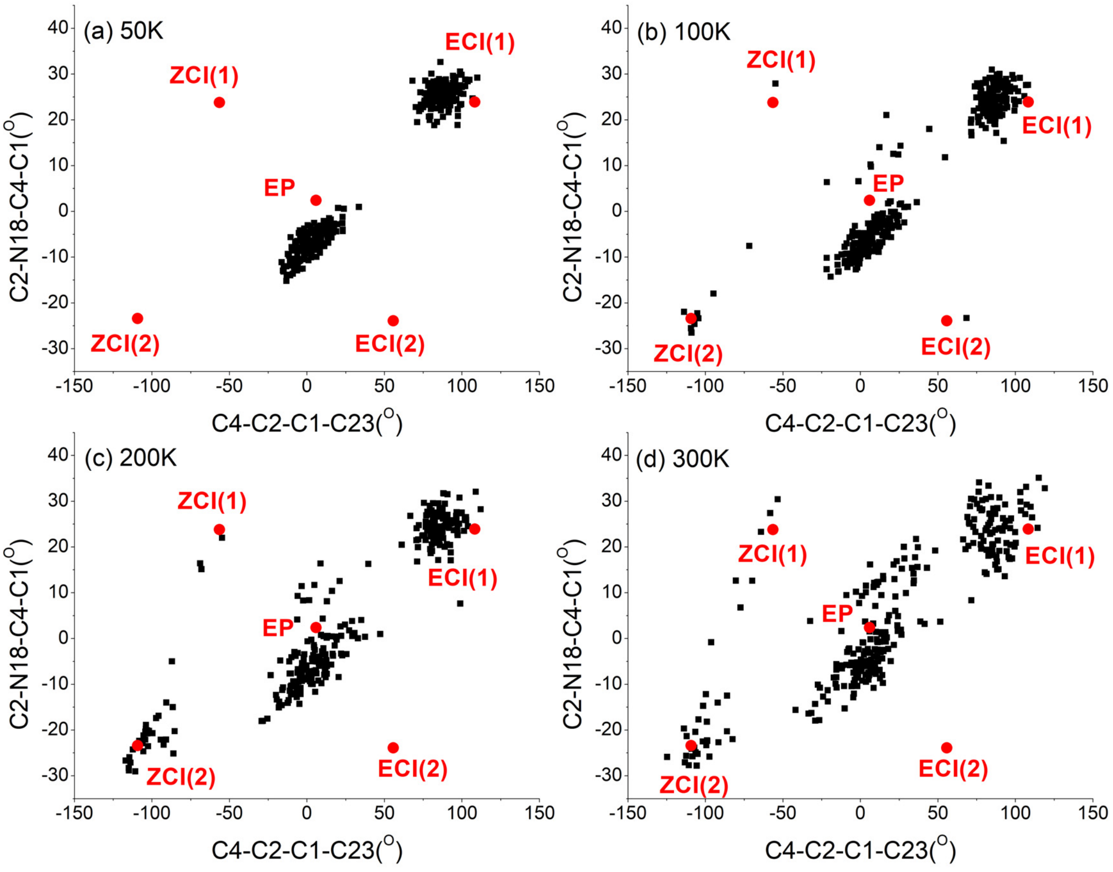

Figure S5. Distribution of the C4-C2-C1-C23 and C2-N18-C4-C1 dihedral angles at the hopping events of all trajectories starting from the *EP* structure of DDPY at (a) 50 K, (b) 100 K, (c) 200 K and (d) 300 K, respectively. The corresponding points of ground state *EP* isomer, conical intersection *ECI(1)*, *ECI(2)*, *ZCI(1)* and *ZCI(2)* are also presented in this figure.

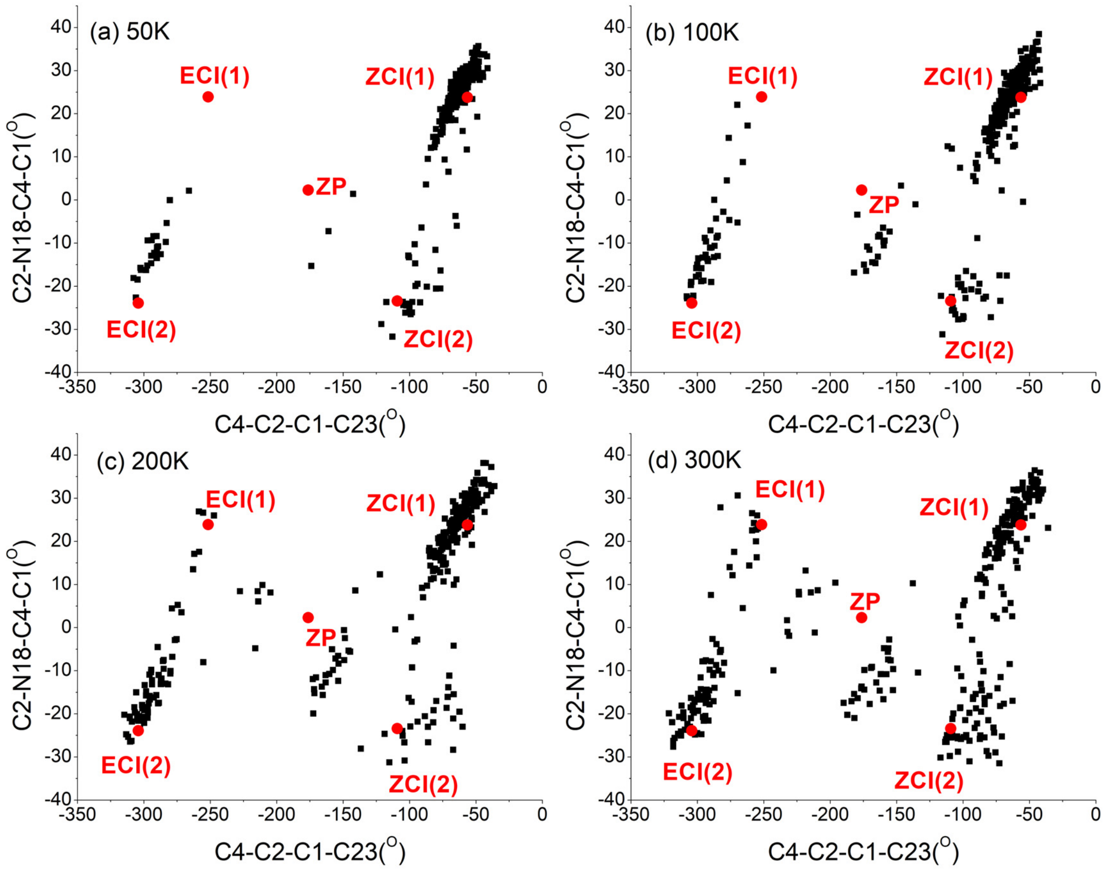

Figure S6. Distribution of the C4-C2-C1-C23 and C2-N18-C4-C1 dihedral angles at the hopping events of all trajectories starting from the *ZP* structure of DDPY at (a) 50 K, (b) 100 K, (c) 200 K and (d) 300 K, respectively. The corresponding points of ground state *ZP* isomer, conical intersection *ECI(1)*, *ECI(2)*, *ZCI(1)* and *ZCI(2)* are also presented in this figure.

5. Time-dependent evolution of geometrical parameters in several typical trajectories of two-stroke LDMRM DDPY at 50 K

In order to explore the reaction dynamics of two-stroke LDMRM DDPY in detail, time dependent evolution of geometrical parameters in five typical trajectories for both *EP→ZP* and *ZP→EP* photoisomerization processes are presented in the following.

Time dependence of central bond length C1-C2, central dihedral angle C4−C2−C1−C23, side dihedral angle C2−C1−C23−N24 and pyramid dihedral angle C2−N18−C4−C1 in five typical trajectories (named as trajectory 1 to trajectory 5) of *EP→ZP* photoisomerization process are shown in Figure 6 in the main text and Figure S7 to Figure S10. The S1→S0 hopping time and correponding geometrical parameters of reaction product *ZP* isomer are also shown in the figures.

Time dependence of central bond length C1-C2, central dihedral angle C4−C2−C1−C23, side dihedral angle C2−C1−C23−N24 and pyramid dihedral angle C2−N18−C4−C1 in five typical trajectories (named as trajectory 1 to trajectory 5) of *ZP→EP* photoisomerization process are shown in Figure 9 in the main text and Figure S11 to Figure S14. The S1→S0 hopping time and correponding geometrical parameters of reaction product *EP* isomer are also shown in the figures.

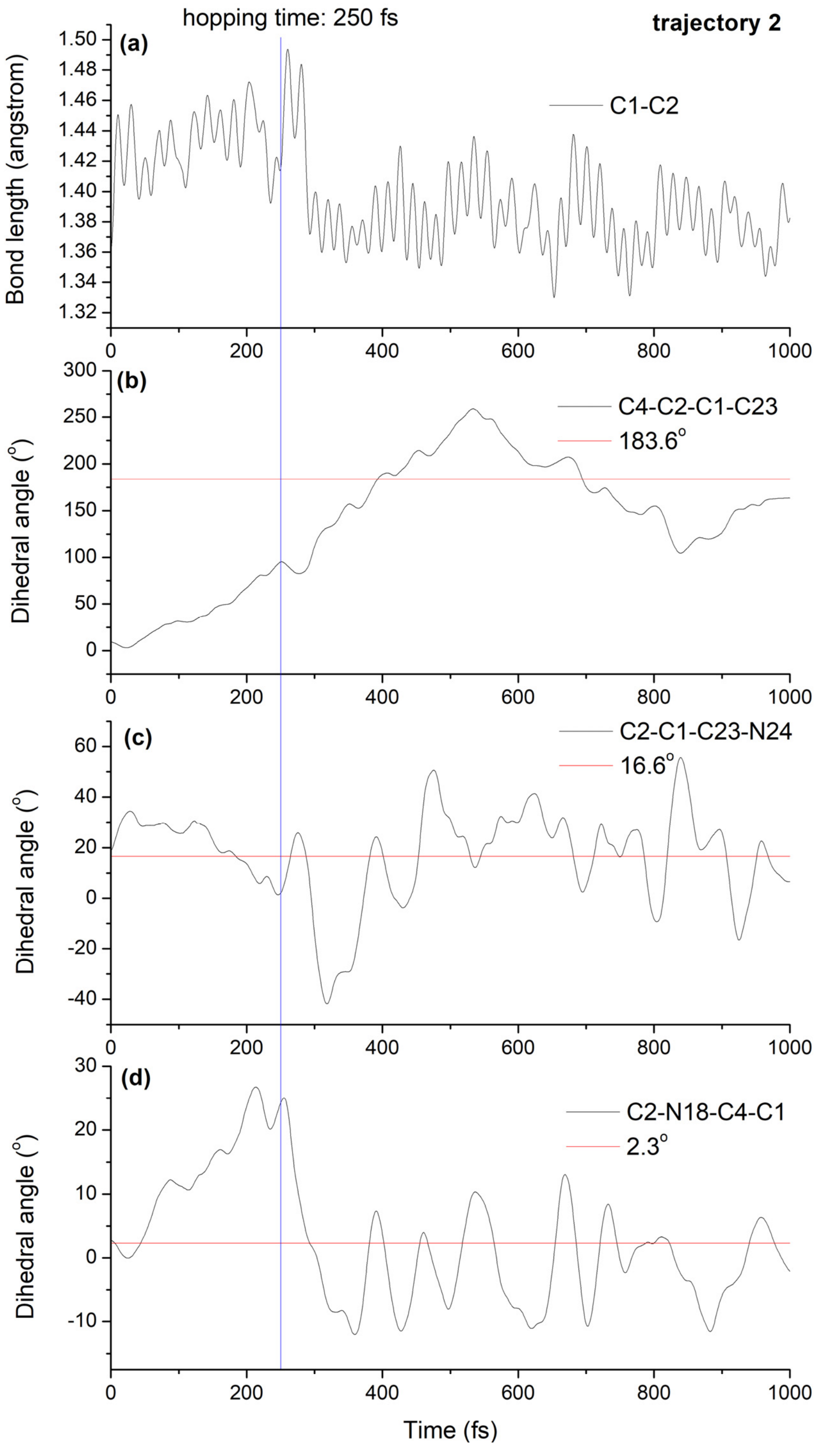

Figure S7. Time dependence of (a) central bond length C1-C2, (b) central dihedral angle C4−C2−C1−C23, (c) side dihedral angle C2−C1−C23−N24 and (d) pyramid dihedral angle C2−N18−C4−C1 in a representative trajectory (**named as trajectory 2**) of *EP*→*ZP* photoisomerization process. The $S_1 \rightarrow S_0$ hopping time (blue line) and correponding geometrical parameters of reaction product ZP isomer (red lines) are also shown in the figure.

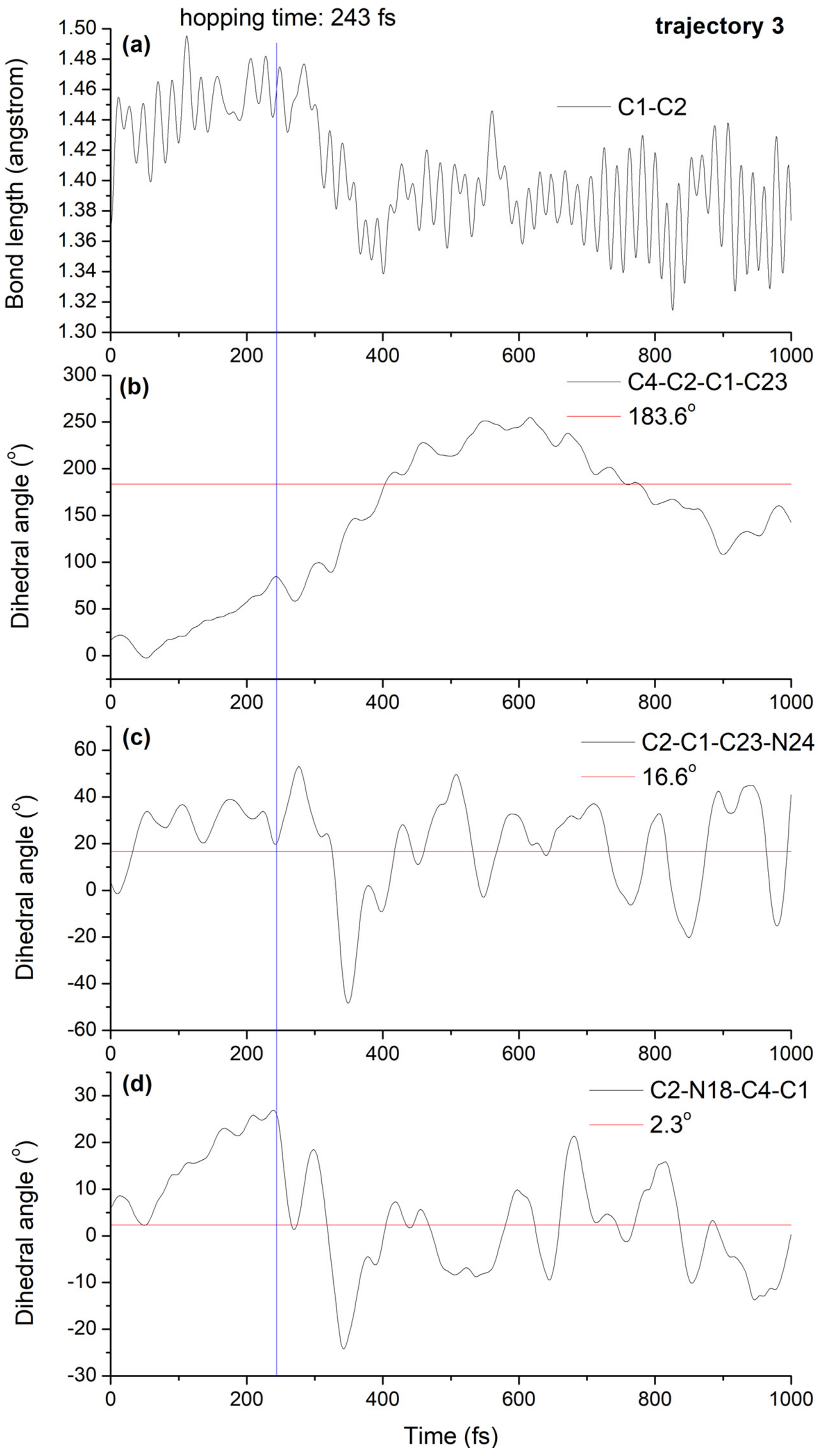

Figure S8. Time dependence of (a) central bond length C1-C2, (b) central dihedral angle C4−C2−C1−C23, (c) side dihedral angle C2−C1−C23−N24 and (d) pyramid dihedral angle C2−N18−C4−C1 in a representative trajectory (**named as trajectory 3**) of *EP*→*ZP* photoisomerization process. The $S_1 \rightarrow S_0$ hopping time (blue line) and correponding geometrical parameters of reaction product ZP isomer (red lines) are also shown in the figure.

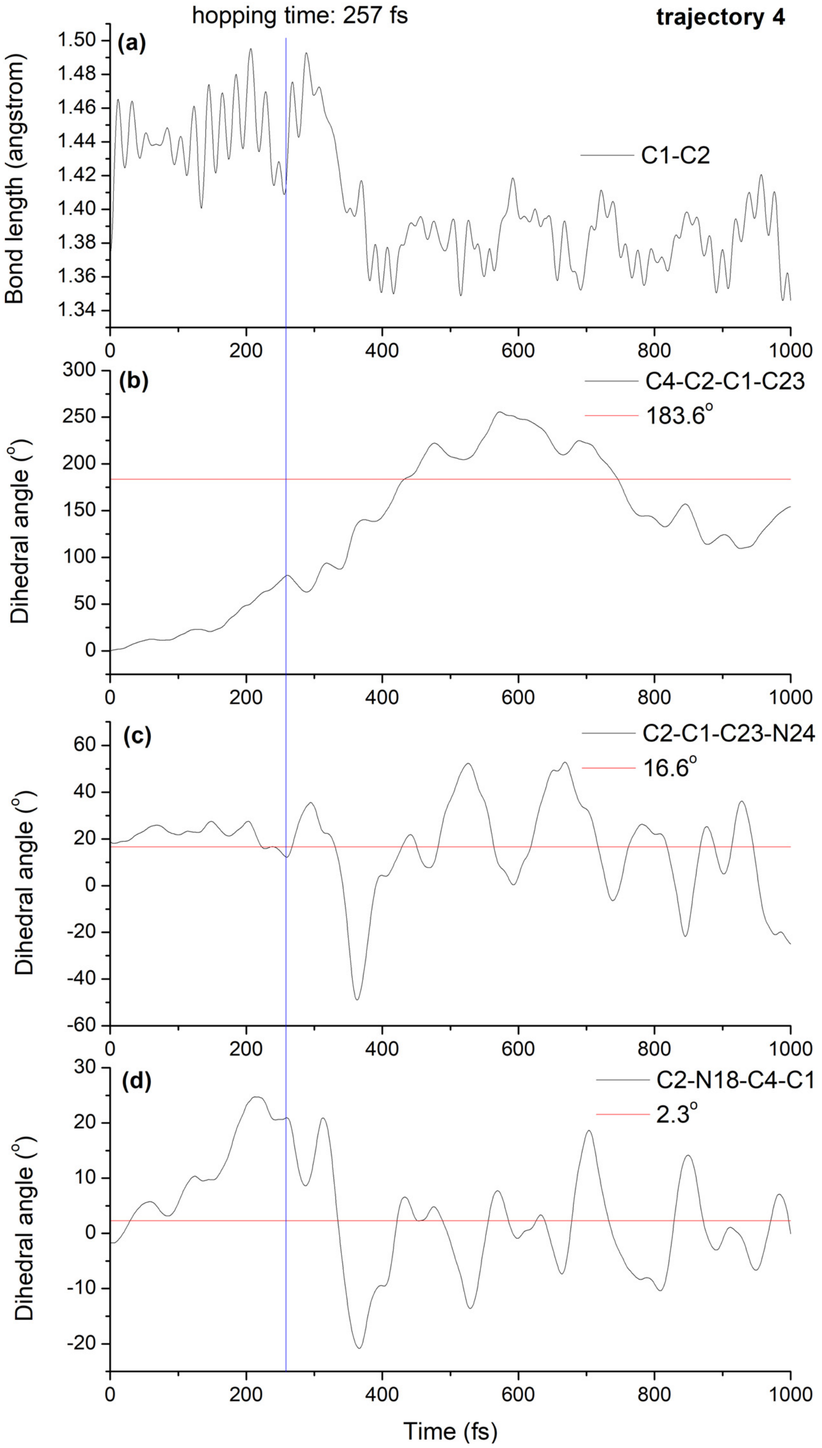

Figure S9. Time dependence of (a) central bond length C1-C2, (b) central dihedral angle C4−C2−C1−C23, (c) side dihedral angle C2−C1−C23−N24 and (d) pyramid dihedral angle C2−N18−C4−C1 in a representative trajectory (**named as trajectory 4**) of *EP*→*ZP* photoisomerization process. The $S_1 \rightarrow S_0$ hopping time (blue line) and correponding geometrical parameters of reaction product ZP isomer (red lines) are also shown in the figure.

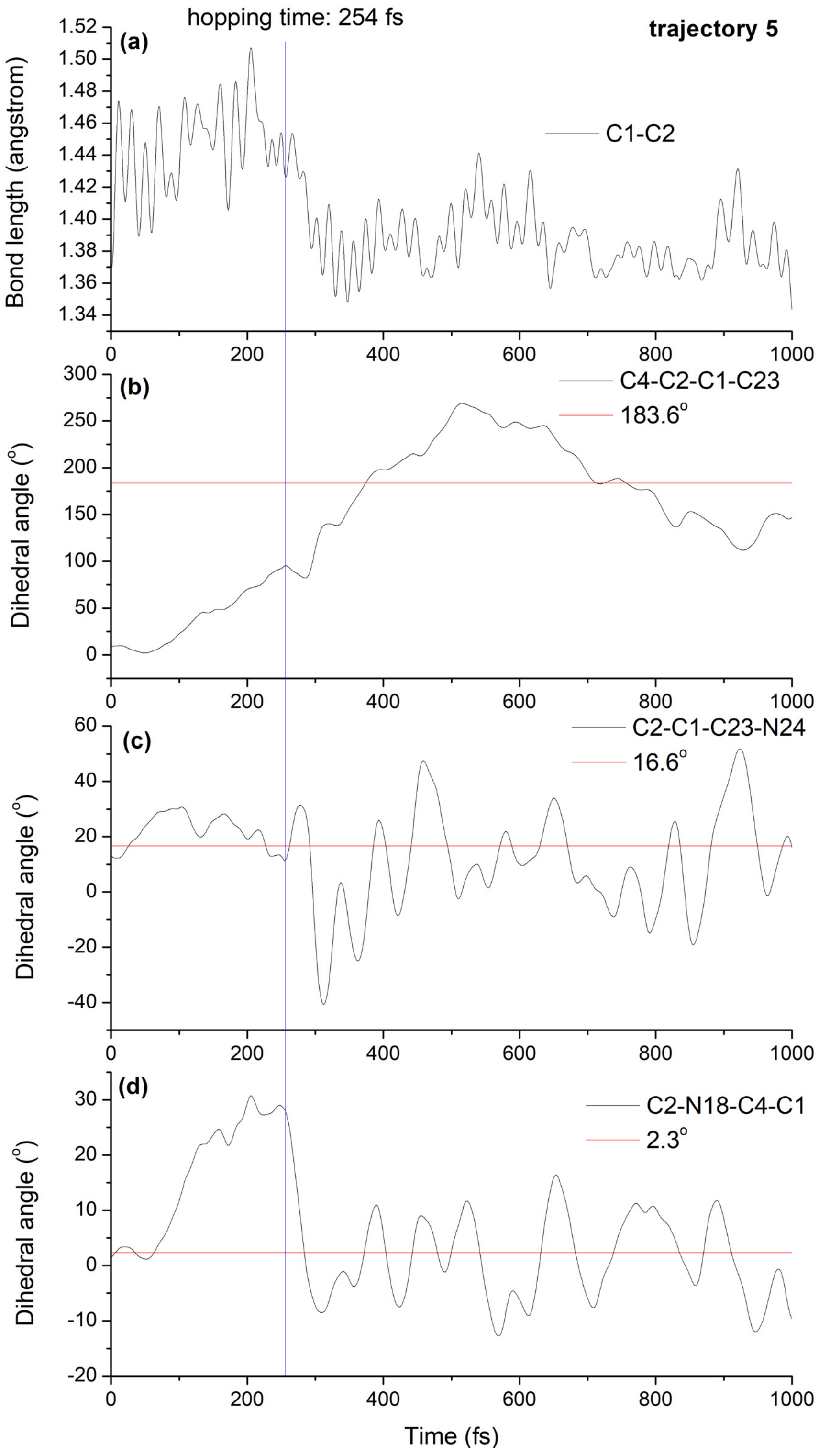

Figure S10. Time dependence of (a) central bond length C1-C2, (b) central dihedral angle C4−C2−C1−C23, (c) side dihedral angle C2−C1−C23−N24 and (d) pyramid dihedral angle C2−N18−C4−C1 in a representative trajectory (**named as trajectory 5**) of *EP*→*ZP* photoisomerization process. The S_1→S_0 hopping time (blue line) and correponding geometrical parameters of reaction product ZP isomer (red lines) are also shown in the figure.

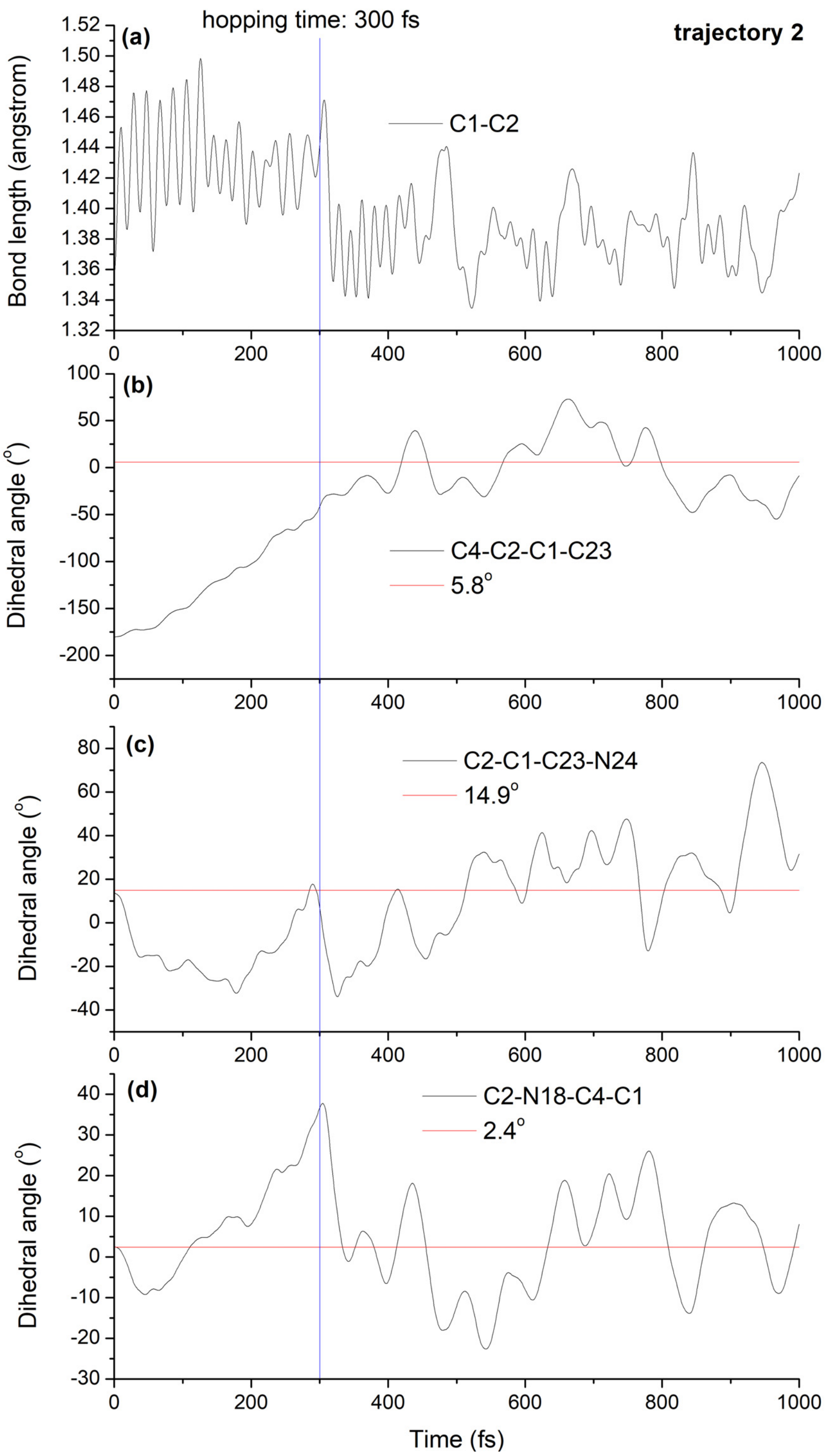

Figure S11. Time dependence of (a) central bond length C1-C2, (b) central dihedral angle C4−C2−C1−C23, (c) side dihedral angle C2−C1−C23−N24 and (d) pyramid dihedral angle C2−N18−C4−C1 in a representative trajectory (**named as trajectory 2**) of *ZP*→*EP* photoisomerization process. The $S_1 \rightarrow S_0$ hopping time (blue line) and correponding geometrical parameters of reaction product EP isomer (red lines) are also shown in the figure.

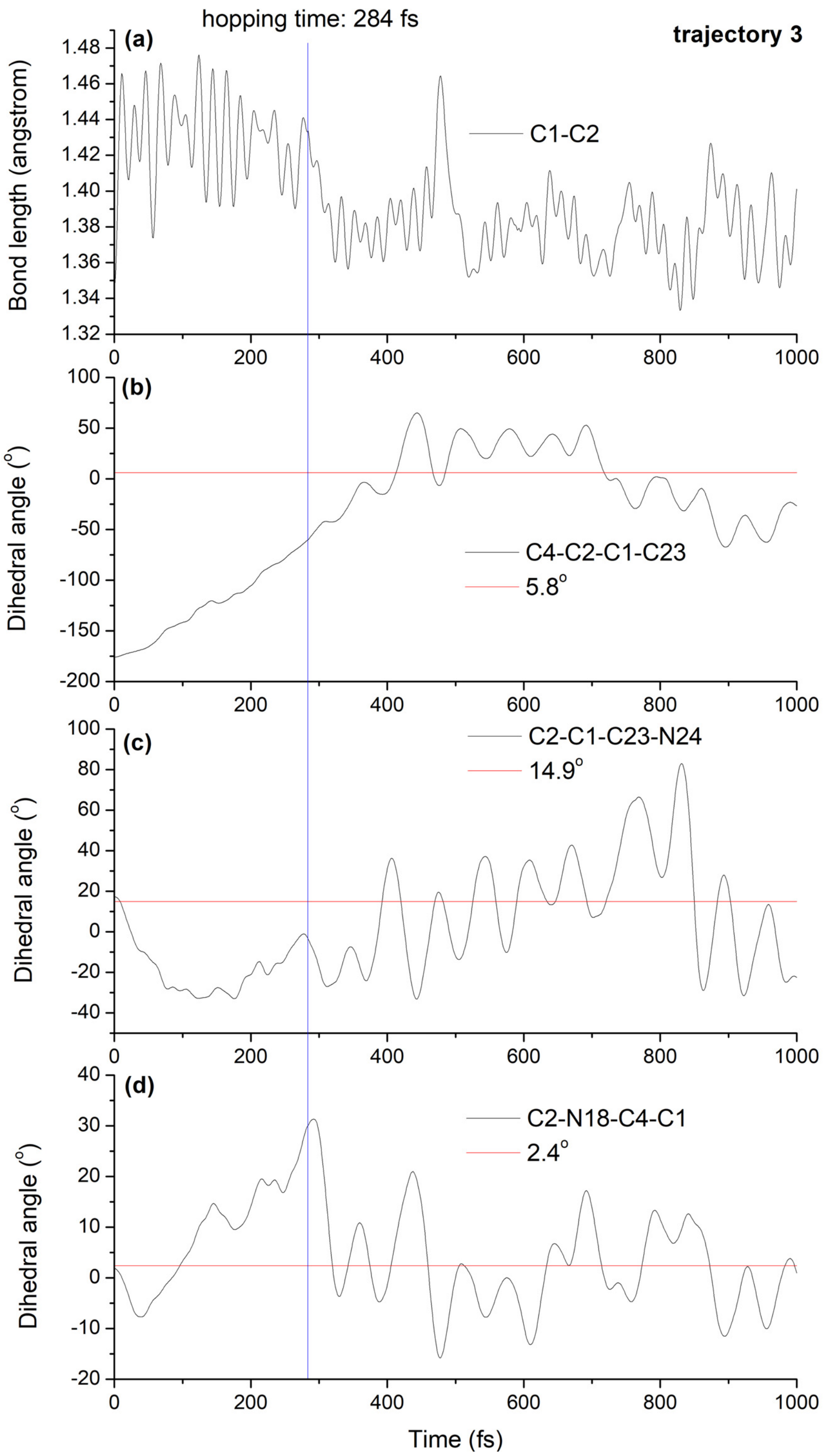

Figure S12. Time dependence of (a) central bond length C1-C2, (b) central dihedral angle C4−C2−C1−C23, (c) side dihedral angle C2−C1−C23−N24 and (d) pyramid dihedral angle C2−N18−C4−C1 in a representative trajectory (**named as trajectory 3**) of *ZP*→*EP* photoisomerization process. The $S_1 \rightarrow S_0$ hopping time (blue line) and correponding geometrical parameters of reaction product EP isomer (red lines) are also shown in the figure.

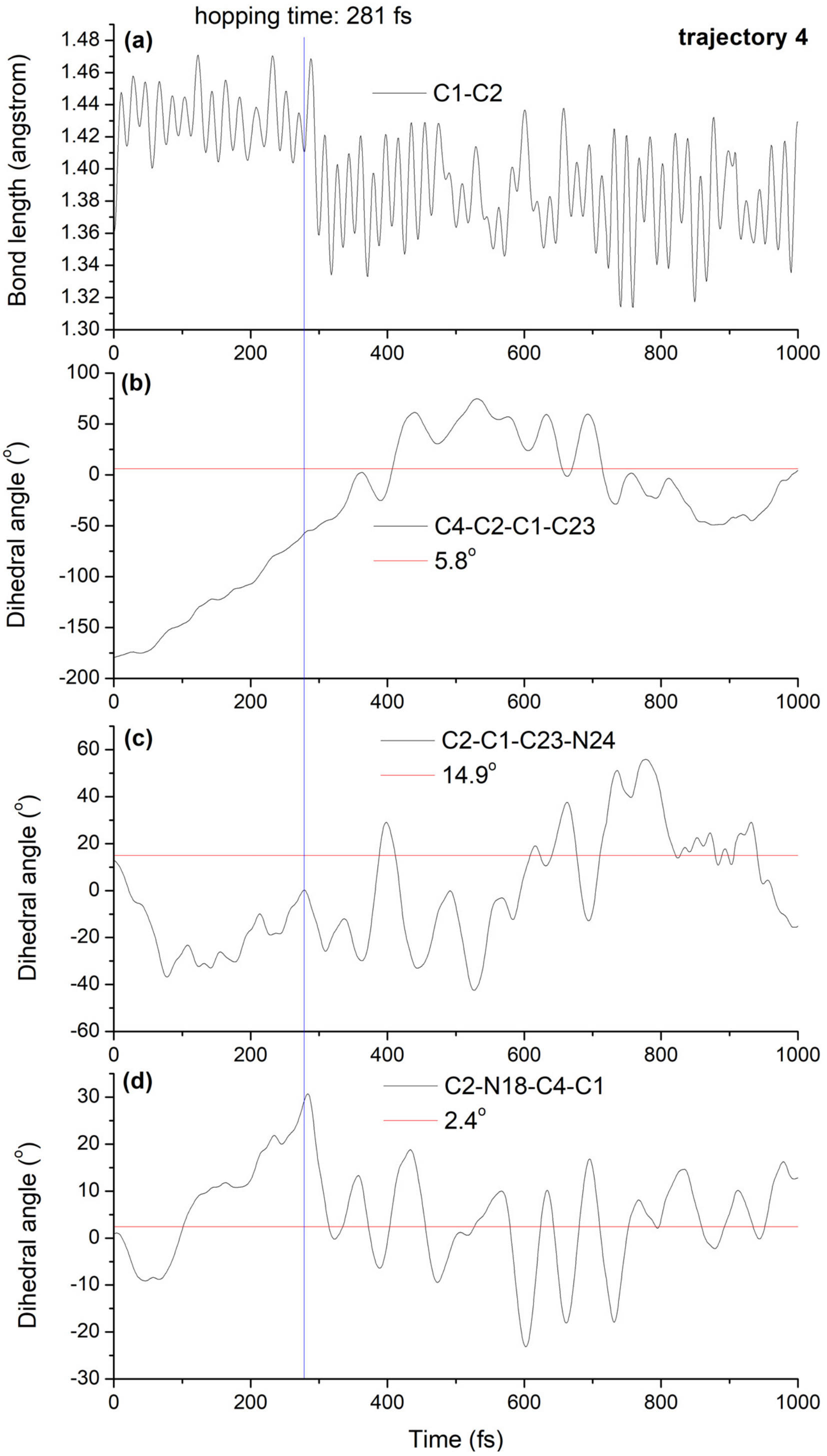

Figure S13. Time dependence of (a) central bond length C1-C2, (b) central dihedral angle C4−C2−C1−C23, (c) side dihedral angle C2−C1−C23−N24 and (d) pyramid dihedral angle C2−N18−C4−C1 in a representative trajectory (**named as trajectory 4**) of *ZP*→*EP* photoisomerization process. The $S_1 \rightarrow S_0$ hopping time (blue line) and correponding geometrical parameters of reaction product EP isomer (red lines) are also shown in the figure.

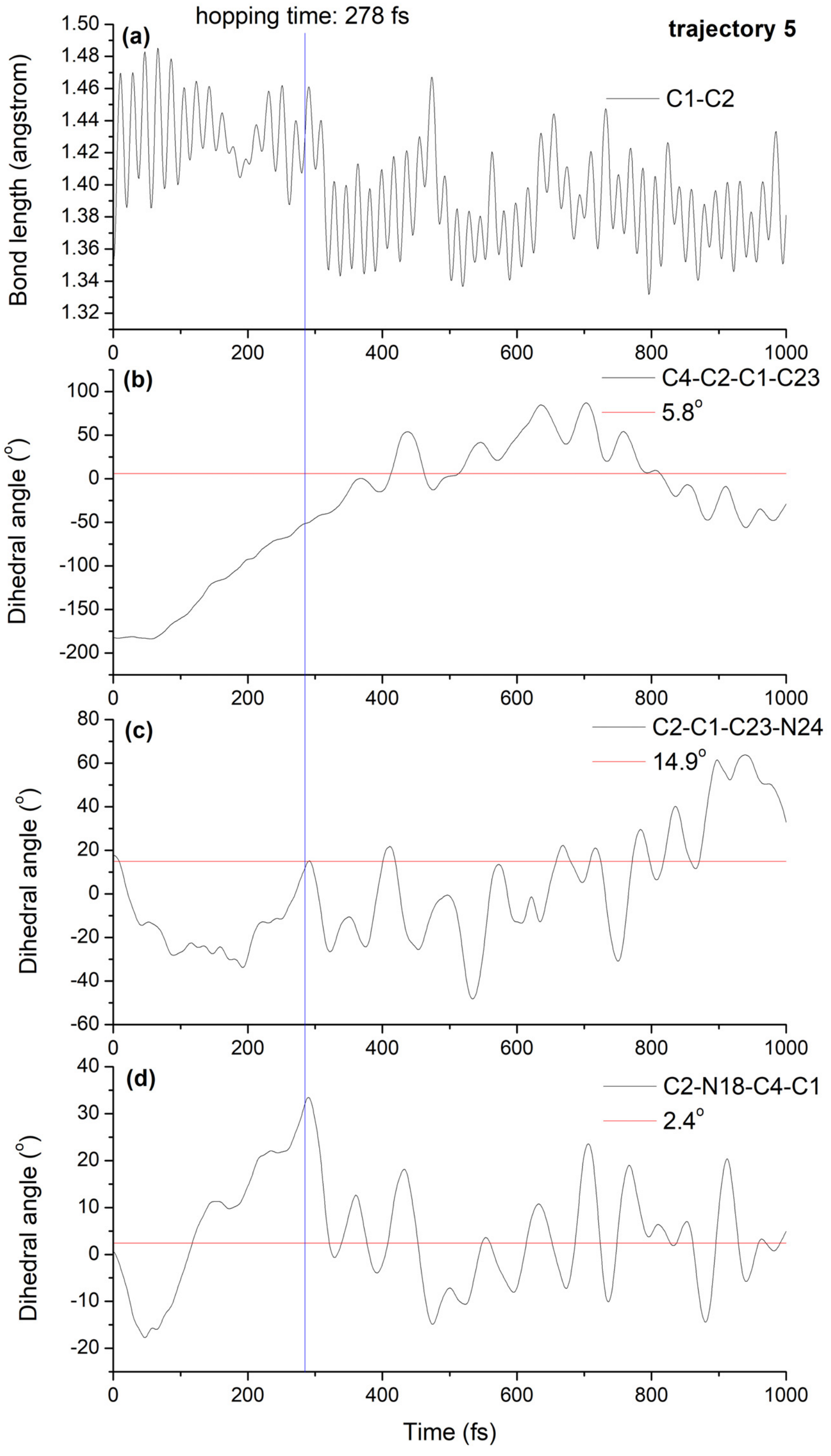

Figure S14. Time dependence of (a) central bond length C1-C2, (b) central dihedral angle C4−C2−C1−C23, (c) side dihedral angle C2−C1−C23−N24 and (d) pyramid dihedral angle C2−N18−C4−C1 in a representative trajectory (**named as trajectory 5**) of *ZP*→*EP* photoisomerization process. The $S_1 \rightarrow S_0$ hopping time (blue line) and correponding geometrical parameters of reaction product EP isomer (red lines) are also shown in the figure.

6. Cartesian coordinates for several structures of two-stroke LDMRM DDPY optimized with the OM2/MRCI method

Unit of the Cartesian coordinate below is angstrom.

(1) Four ground state local minima geometries for the two-stroke LDMRM DDPY.

ZP

C	0.0893762508	0.3620858215	-0.1085121837
C	1.3018518701	-0.2670012988	-0.1210517390
C	2.9011202654	-1.8867829334	-0.1131597058
C	2.6488832511	0.3938955251	-0.0664164500
C	-1.5478394947	2.1177396013	-0.6463358178
C	-0.0796779871	1.8767451256	-0.2010820730
C	0.1720299677	2.4687510081	1.1733342030
O	2.8413092985	1.6087052448	-0.0224678739
H	-2.0298117981	2.9285208344	-0.0590010852
H	-1.6014351728	2.3731913106	-1.7281157869
H	0.6440186114	2.2959340510	-0.9451527438
H	1.2123811148	2.2967627436	1.4697282595
H	-0.4998894641	2.0190598204	1.9208381760
H	-0.0085722278	3.5558083397	1.1446144235
H	3.3139602041	-2.8976448254	-0.1403833201
C	3.6113197689	-0.7087659653	-0.0501648093
H	4.6782241738	-0.5888649902	-0.0321730501
N	1.5352250151	-1.6583242008	-0.0756235257
H	0.8833339377	-2.3194599231	-0.4687696113
C	-3.2688448578	-1.1340719276	0.0198069461
C	-3.4762779429	0.2113932652	-0.3390112643
C	-2.1971814924	0.8051993814	-0.3940047636
C	-1.2405927026	-0.1972145393	-0.0889837721
N	-1.9095234409	-1.3820516339	0.1738192121
H	-4.4288861463	0.6777420077	-0.5288534395
C	-1.3599664093	-2.6162777946	0.6649212611
H	-0.9350969846	-3.2187585320	-0.1735777188
H	-0.5662776171	-2.4166828565	1.4117481707
H	-2.1516542672	-3.2208752246	1.1532816152
H	-4.0281641164	-1.8908855244	0.1800678920

ZM

C	-0.0715914345	-0.3582062918	-0.0235703273
C	-1.2767650849	0.2849162101	0.0494780277
C	-2.8681209245	1.9064772215	0.1986168515
C	-2.6120084880	-0.3241785246	-0.2736319991
C	1.5422624532	-2.2085020989	-0.3272419871
C	0.0369570543	-1.8769890717	-0.1442195298
C	-0.4904911241	-2.4993298917	1.1364527160
O	-2.7949375760	-1.4917420233	-0.6182446650
H	1.9033594044	-2.9196962166	0.4463738871
H	1.7424679151	-2.6376764570	-1.3342432075
H	-0.5652486942	-2.2214754606	-1.0223605862
H	-1.5568110771	-2.2735565279	1.2546100190
H	0.0597928370	-2.1203864855	2.0125193449
H	-0.3698974460	-3.5937255982	1.0897497968
H	-3.2813071130	2.9001828689	0.3856655657
C	-3.5681472901	0.7797592233	-0.1685843587
H	-4.6275640311	0.6886508587	-0.3194185575
N	-1.5038687451	1.6639767277	0.2492219332
H	-0.9069198453	2.2118866606	0.8513466280
C	3.3231618628	1.0650150864	-0.0764224043
C	3.4998851575	-0.3226767626	-0.2265788629
C	2.2087148675	-0.8889762957	-0.1887153819
C	1.2734681171	0.1631509337	-0.0054197235
N	1.9709688529	1.3614826516	0.0508681477
H	4.4413081939	-0.8337379428	-0.3430648837
C	1.4468276179	2.6967592897	0.1243211858
H	0.6874878652	2.8598316541	-0.6663213596
H	0.9916544883	2.8845150620	1.1240089541
H	2.2611475871	3.4358586191	-0.0206408249
H	4.0979872820	1.8253089179	-0.0658537628

EP

C	0.0775405063	0.5562760863	-0.0442752001
C	1.3974905410	0.1694082794	-0.0286349209
C	3.6611900909	0.5191346794	-0.0919806338
C	2.0970806775	-1.1597585179	-0.1016813422
C	-1.7889229445	2.0323767349	-0.7255727502
C	-0.3276767807	2.0250453259	-0.2101132227
C	-0.2385026558	2.7078338996	1.1432726064
O	1.5616445958	-2.2707390053	-0.1804654428
H	-2.4174269318	2.7854874722	-0.2010441537
H	-1.8194641686	2.2306689098	-1.8176335921
H	0.3498935735	2.5374668018	-0.9533135214

H	0.7853158324	2.6549772540	1.5485518721
H	-0.9168970398	2.2174853078	1.8536807357
H	-0.5243672763	3.7670676329	1.0502360641
H	4.5950422722	1.0838656675	-0.1118151949
C	3.5224014027	-0.8537565297	-0.1277296218
H	4.3041434601	-1.5885635726	-0.1597417890
N	2.4331256512	1.1322391523	-0.0563177488
H	2.3036861959	2.1294821921	0.0063151138
C	-3.0092743758	-1.4168190170	-0.0167723870
C	-3.4026667546	-0.1314822828	-0.4430916220
C	-2.2315970187	0.6468751285	-0.4359238214
C	-1.1478461266	-0.1883673993	-0.0317957856
N	-1.6474251017	-1.4530690269	0.2162332012
H	-4.4001807049	0.1744718322	-0.7114683555
C	-0.9695983535	-2.5874321026	0.7984228677
H	-1.5119658156	-3.5212366457	0.5375623334
H	0.0626695162	-2.6541901948	0.4307368691
H	-0.9669202805	-2.4896629663	1.9076269420
H	-3.6474548135	-2.2830991678	0.0941162625

EM

C	0.0584776882	0.5472162002	-0.0702957492
C	1.3597118693	0.1065146727	-0.0949844230
C	3.6158372090	0.2986894677	-0.4217027696
C	2.0130606150	-1.1825319770	0.2843416801
C	-1.8076531082	2.1737553902	-0.2780197877
C	-0.2690925914	2.0345653700	-0.1792043327
C	0.2571100131	2.7420940891	1.0575487078
O	1.4692781034	-2.1490975021	0.8299883620
H	-2.2173207480	2.7742368045	0.5635967453
H	-2.1154107590	2.6485696848	-1.2387753529
H	0.2143721360	2.4610258726	-1.1094333023
H	1.3421009277	2.6092029487	1.1633889130
H	-0.2299152690	2.3356173438	1.9545728836
H	0.0375688913	3.8195757591	0.9897319587
H	4.5538815923	0.7668836374	-0.7206364742
C	3.4401792567	-0.9904702534	0.0335719467
H	4.1896571847	-1.7407445883	0.1899542032
N	2.4214796852	0.9892520367	-0.4160337922
H	2.2869139025	1.8230024360	-0.9700312401
C	-3.0780893749	-1.3235110703	-0.2731185995
C	-3.4613976683	0.0321917157	-0.3342282318
C	-2.2698425894	0.7697641654	-0.2064726535

C	-1.1919273410	-0.1550109952	-0.0363970378
N	-1.7115761150	-1.4344919784	-0.1047251840
H	-4.4618609619	0.4076257917	-0.4686371106
C	-1.0245412306	-2.7039666051	-0.0818622460
H	-1.6760678342	-3.4748544834	0.3814847997
H	-0.7958274227	-3.0244663130	-1.1241671422
H	-0.0879782337	-2.6322291855	0.4862360515
H	-3.7327406623	-2.1815838492	-0.3332449479

(2) Two transition state geometries at ground state for the two-stroke LDMRM DDPY.

EM-EP-TS

C	0.0602173059	0.5194646108	-0.0540486720
C	1.3641674105	0.0981949474	-0.1764245803
C	3.6026715893	0.3860561552	-0.5906920701
C	2.0678402095	-1.2123260170	-0.0013456591
C	-1.8039624670	2.1499466133	-0.2866067951
C	-0.2730229322	2.0119845824	-0.1167599496
C	0.1861217227	2.6553825562	1.1802497288
O	1.5740245910	-2.2714990227	0.4003884925
H	-2.2471477478	2.8140292805	0.4860615615
H	-2.0663036893	2.5414138137	-1.2938256658
H	0.2500264727	2.4901602492	-0.9977109376
H	1.2629165631	2.5045990734	1.3398777913
H	-0.3545025972	2.2106314653	2.0265335699
H	-0.0203733188	3.7372748022	1.1543679907
H	4.5161678473	0.9126949162	-0.8684723739
C	3.4749968672	-0.9535304513	-0.2896745333
H	4.2474882140	-1.6964047765	-0.2566385080
N	2.3935974634	1.0315880355	-0.4627443157
H	2.2294573601	1.9514146668	-0.8380770956
C	-3.1105981734	-1.3212985177	-0.0136244217
C	-3.4763711702	0.0318508207	-0.1606747406
C	-2.2696857449	0.7531530404	-0.1299649271
C	-1.1947192354	-0.1735234515	0.0419076038
N	-1.7390163145	-1.4443830281	0.0988950564
H	-4.4749107921	0.4154332251	-0.2854222810
C	-1.1011752091	-2.7262086221	0.2871938679
H	-1.2681212585	-3.3635445034	-0.6112553277
H	-0.0236135024	-2.6127413804	0.4496993173
H	-1.5509010235	-3.2408218777	1.1650031556

H	-3.7787077330	-2.1723651063	0.0058559156

ZM-ZP-TS

C	0.0984653345	0.3521391395	-0.0416832036
C	1.3065650572	-0.2895860586	-0.0474549373
C	2.9011527319	-1.9186695335	-0.1010081430
C	2.6612246160	0.3624133141	-0.1638965204
C	-1.4858407548	2.1654121978	-0.5593372385
C	-0.0268600639	1.8740436990	-0.1183685348
C	0.2452829956	2.4618393194	1.2546787304
O	2.8569128691	1.5762749360	-0.2322875571
H	-1.9477699540	2.9811945652	0.0364646981
H	-1.5273863626	2.4326891545	-1.6375399231
H	0.7111728236	2.2758203677	-0.8571947632
H	1.2703321487	2.2295169424	1.5674133289
H	-0.4613278027	2.0713177971	2.0036707552
H	0.1358719385	3.5584198979	1.2083845843
H	3.3060919141	-2.9334565025	-0.1004986420
C	3.6158011970	-0.7426131024	-0.1983356286
H	4.6787782783	-0.6326057947	-0.2960398159
N	1.5507162873	-1.6728179356	0.0109298532
H	0.8534327929	-2.3952916171	0.0505085716
C	-3.3099063844	-1.0554524705	-0.0456949280
C	-3.4688841987	0.3074524297	-0.3536724970
C	-2.1723474204	0.8656102836	-0.3395907623
C	-1.2484326258	-0.1717320701	-0.0490392006
N	-1.9613359979	-1.3491854867	0.1301474937
H	-4.3995994386	0.8031921128	-0.5755173212
C	-1.4870062235	-2.6527948736	0.4988558544
H	-0.9569234181	-3.1372075985	-0.3530188813
H	-0.8070132651	-2.5908359560	1.3752013092
H	-2.3446044733	-3.2983731326	0.7786238629
H	-4.0914462206	-1.8011057770	0.0636663058

(3) Four different S_1/S_0 CIs for the two-stroke LDMRM DDPY.

ZCI(1)

C	0.0402205577	0.7864486499	-0.2136287988
C	1.4231985111	0.4891027644	-0.1761566738
C	3.1644500035	-0.7670254984	-0.9160756791
C	1.8287497971	-0.3981359857	0.9265675319
C	-2.0913238380	2.0292169052	0.0471280584

C	-0.5438202136	2.1533472643	0.0989775069
C	-0.0575143214	2.5429713915	1.4848407297
O	1.3081680473	-0.4716021000	2.0602721254
H	-2.5648751073	2.3907143718	0.9798608765
H	-2.5117027385	2.5835314478	-0.8177812235
H	-0.1748630389	2.8846289656	-0.6664134128
H	1.0362286129	2.5599775271	1.4915744356
H	-0.3961503864	1.8106219961	2.2271843395
H	-0.4452563783	3.5401724665	1.7486705446
H	3.9649113072	-1.0519140731	-1.5662015256
C	3.0315504061	-1.0843163627	0.4192296878
H	3.6006330079	-1.7992192384	0.9919911033
N	2.1243350415	0.0388999949	-1.3469736475
H	2.3720586443	0.7766123253	-2.0120521615
C	-2.6414532858	-1.6530437260	-0.2947358091
C	-3.3103256288	-0.4009251162	-0.1440917393
C	-2.3114487407	0.5673495713	-0.1166996103
C	-1.0508621190	-0.1090548611	-0.2798783895
N	-1.2933261459	-1.4723842306	-0.3814102004
H	-4.3742669569	-0.2719152347	-0.0544160458
C	-0.2589714954	-2.4839645652	-0.4338953566
H	0.3983542720	-2.3028658366	-1.3064169020
H	0.3490431215	-2.4304521976	0.4857625762
H	-0.7126104536	-3.4917835647	-0.5211153899
H	-3.1236654487	-2.6267907263	-0.3424923734

ZCI(2)

C	0.0638690846	0.5172496005	-0.1817969065
C	1.3114173512	-0.1617018065	-0.2128040447
C	3.5389961412	-0.1090957003	-0.6816562983
C	2.0514847089	-0.1861968301	1.0759271842
C	-1.8587410247	2.0429403939	0.2398488394
C	-0.3046033797	1.9541073316	0.1954529978
C	0.3126432953	2.2965621965	1.5365950460
O	1.5409964590	-0.3660470959	2.1977469740
H	-2.2204754643	2.3810483251	1.2311699935
H	-2.2457407133	2.7257336808	-0.5454450220
H	0.1027222610	2.6276989575	-0.6063343476
H	1.4051206352	2.2372772020	1.4655314492
H	-0.0190323234	1.5884409544	2.3029756585
H	0.0253261837	3.3199520362	1.8284662337
H	4.4177744904	-0.1840301824	-1.2917069325
C	3.4696174674	-0.2795767289	0.6856358443

H	4.2867977413	-0.3195433455	1.3868933934
N	2.2866714805	0.1085755305	-1.2236840405
H	2.0997962605	-0.2653420980	-2.1549526068
C	-2.9330556575	-1.4986792309	-0.3331769231
C	-3.4198338707	-0.1821150637	-0.0760375967
C	-2.2994117877	0.6450175903	-0.0186128397
C	-1.1540283581	-0.1820407804	-0.2692749551
N	-1.5723139298	-1.4912020106	-0.4517579840
H	-4.4547437303	0.0807247310	0.0577391263
C	-0.6501623250	-2.5906330671	-0.6236592325
H	-0.7075177861	-2.9838405534	-1.6631705470
H	0.3695400193	-2.2203116540	-0.4202820565
H	-0.8950066436	-3.4090180941	0.0797277720
H	-3.5426731914	-2.3930475801	-0.4274960963

ECI(1)

C	0.0649901749	0.4120085574	0.0497428681
C	1.2840509457	-0.2159242074	0.4210166696
C	3.4835263467	-0.0208270802	0.9705963505
C	2.0895845202	-0.6897986968	-0.7344541628
C	-1.7324563825	1.7309298044	-1.0502247821
C	-0.1914135346	1.5955926648	-0.8816888294
C	0.4316354434	2.8466543203	-0.2963525881
O	1.6314096091	-1.1726209298	-1.7869736369
H	-2.1134307229	2.6443991122	-0.5475010679
H	-2.0286510812	1.7477751790	-2.1180636256
H	0.2855953344	1.3404042537	-1.8606105289
H	1.5006052986	2.6793556190	-0.1230832903
H	-0.0472393469	3.1060760125	0.6609885560
H	0.3069819226	3.6881122205	-0.9947361365
H	4.3278420083	0.1099649696	1.6187754424
C	3.4801087181	-0.6531142040	-0.2561421423
H	4.3295275995	-0.9402518638	-0.8534585209
N	2.2130613419	0.3858606838	1.3276788274
H	1.9668046128	0.3732253999	2.3183956524
C	-3.0360280262	-1.3661257790	0.6042413055
C	-3.4271942537	-0.2176672023	-0.1456073054
C	-2.2627649899	0.5135554159	-0.3767319717
C	-1.1878963654	-0.1958658155	0.2558808932
N	-1.6905454242	-1.3451438268	0.8456044349
H	-4.4311394603	0.0011459188	-0.4654309978
C	-0.8543155802	-2.3324976077	1.4878618915
H	-1.0725565293	-3.3395689088	1.0848226082

H	0.2002403242	-2.0781254750	1.2885109318
H	-1.0377435147	-2.3395986073	2.5853617835
H	-3.6988692290	-2.1537794537	0.9513941410

ECI(2)

C	0.0397354602	0.7037100638	-0.0197350350
C	1.4064015784	0.3964998560	0.1784133065
C	3.0233384025	-0.5418187132	1.4679574925
C	1.8561998695	-0.8314129265	-0.4986859452
C	-2.0035661595	1.8120777182	-0.8880310497
C	-0.4522340425	1.8830150656	-0.8410398840
C	0.0462245121	3.1718278316	-0.2172995376
O	1.4228219856	-1.2934096114	-1.5759462884
H	-2.4611093741	2.6268668923	-0.2902923723
H	-2.3876890337	1.8568589696	-1.9255982390
H	-0.0266433730	1.7580532141	-1.8666360547
H	1.1392563993	3.1414761810	-0.1488234990
H	-0.3744305157	3.3034474702	0.7921430454
H	-0.2532831164	4.0275021582	-0.8406341575
H	3.7553541384	-0.5892780538	2.2466446050
C	2.9837929317	-1.3093950753	0.3231986449
H	3.5621319363	-2.1905048190	0.0936574347
N	1.9897202824	0.3790272376	1.4917789209
H	2.2165340112	1.3006159079	1.8754621597
C	-2.7444225633	-1.5322344488	0.6083750032
C	-3.3401111866	-0.4012056451	-0.0275785478
C	-2.3015504167	0.4914881569	-0.2718096475
C	-1.0934199974	-0.0993627020	0.2422671263
N	-1.4049937868	-1.3437426041	0.7733226615
H	-4.3834017783	-0.3028069712	-0.2690207950
C	-0.4261410414	-2.2884942201	1.2690878496
H	-0.9317733172	-3.1938448708	1.6611834473
H	0.2541960047	-2.5712628468	0.4474354285
H	0.1674573716	-1.8222595250	2.0789641286
H	-3.2716987256	-2.4271114185	0.9310078861

7. Cartesian coordinates for several structures of two-stroke LDMRM DDPY optimized with the B3LYP/6-31G(d) method

Unit of the Cartesian coordinate below is angstrom.

(1) Four ground state local minima geometries for the two-stroke LDMRM DDPY.

ZP

C	0.09541700	0.35179300	-0.07114400
C	1.30712000	-0.26893400	-0.08480000
C	2.89102300	-1.89015600	-0.26706600
C	2.66043500	0.37413500	-0.09190800
C	-1.51897900	2.11530000	-0.68102100
C	-0.06492500	1.88240000	-0.14520800
C	0.12969100	2.52525100	1.24069400
O	2.90597200	1.58047700	-0.00820000
H	-2.00423500	2.96199600	-0.18112100
H	-1.50624500	2.34713000	-1.75519500
H	0.68972600	2.30078600	-0.81518200
H	1.13989900	2.33494400	1.60834200
H	-0.59893700	2.13211700	1.96009600
H	-0.00926100	3.61072600	1.17254200
H	3.24645300	-2.90671600	-0.39066200
C	3.60561300	-0.73978800	-0.20095800
H	4.68118800	-0.63628900	-0.21516000
N	1.52715900	-1.67116200	-0.11770600
H	0.88443300	-2.26183800	-0.62947300
C	-3.26644700	-1.11467200	-0.01621100
C	-3.46593700	0.20495500	-0.39182800
C	-2.18850600	0.80178200	-0.40997800
C	-1.25156600	-0.16765200	-0.06695600
N	-1.92476400	-1.35365300	0.18322800
H	-4.42387900	0.66002900	-0.60472000
C	-1.39625800	-2.61776500	0.66956100
H	-1.12583100	-3.29139700	-0.15487000
H	-0.51545500	-2.44070000	1.28820500
H	-2.15811600	-3.11367200	1.27613200
H	-3.98435900	-1.90711400	0.14896100

ZM

C	0.08146600	0.34066100	-0.04095800
C	1.29117400	-0.28874500	-0.01150900
C	2.88139600	-1.91465400	0.07972300
C	2.63285800	0.32618700	-0.27780300
C	-1.52933400	2.19447000	-0.41171600
C	-0.02939300	1.87857700	-0.10266400
C	0.41831300	2.52582000	1.22076000
O	2.87286400	1.51320500	-0.51647900
H	-1.92037300	2.97228800	0.25599200
H	-1.65446100	2.57135200	-1.43595000
H	0.63252100	2.24938600	-0.88965300
H	1.46729000	2.30277700	1.42447400
H	-0.19621200	2.17039000	2.05732600
H	0.31091000	3.61525200	1.15955700
H	3.24970400	-2.91905900	0.25480100
C	3.57723000	-0.79253400	-0.22590400
H	4.64116700	-0.70684800	-0.39430400
N	1.51237500	-1.68546900	0.13239500
H	0.96809800	-2.20624600	0.80629200
C	-3.31466200	-1.05628000	-0.08723600
C	-3.49421100	0.30521000	-0.26547900
C	-2.20465800	0.87233100	-0.23115700
C	-1.27774700	-0.14819400	-0.03119200
N	-1.97559600	-1.34713700	0.04695900
H	-4.44366500	0.80265000	-0.40974200
C	-1.47657400	-2.69561200	0.24969800
H	-0.69268600	-2.93127000	-0.47335500
H	-1.09092100	-2.83166700	1.26890400
H	-2.30288300	-3.39557200	0.10950800
H	-4.04401700	-1.85426300	-0.04488100

EP

C	0.09864800	0.52356700	-0.05662800
C	1.41473200	0.15311000	-0.04009400
C	3.67135400	0.47487600	-0.03017700
C	2.09986000	-1.16400100	-0.25984500
C	-1.72866400	2.01150200	-0.78869100
C	-0.29802100	2.01242200	-0.15789300
C	-0.31488000	2.69374100	1.22532300
O	1.56586300	-2.25647300	-0.48864900
H	-2.36243400	2.80159200	-0.36768800
H	-1.67010700	2.19195900	-1.87087700
H	0.41295200	2.53787900	-0.80617500

H	0.65246800	2.62781100	1.73797800
H	-1.05952900	2.21785400	1.87304100
H	-0.57248300	3.75479200	1.12897300
H	4.58023200	1.05614200	0.07196000
C	3.52633700	-0.86234200	-0.23031400
H	4.31028500	-1.59205700	-0.37163400
N	2.45384200	1.11676700	0.00852300
H	2.33981200	2.00304600	0.47652100
C	-3.02104000	-1.39128400	-0.00640600
C	-3.39061000	-0.13081700	-0.45987300
C	-2.20866400	0.62811600	-0.47522500
C	-1.15231200	-0.19144600	-0.06520200
N	-1.67146000	-1.44189400	0.23128400
H	-4.39128800	0.17334200	-0.73566000
C	-1.02735700	-2.54365000	0.93890400
H	-1.65372100	-3.43250000	0.82989400
H	-0.04333600	-2.72375500	0.50547200
H	-0.93148500	-2.30838600	2.00614400
H	-3.63124300	-2.26280200	0.18932500

EM

C	0.06255300	0.49558800	-0.13050000
C	1.36790900	0.07113200	-0.16159500
C	3.61403100	0.30426300	-0.50666500
C	2.06718200	-1.18959000	0.25794900
C	-1.79760600	2.16708900	-0.23512500
C	-0.24473300	2.01683300	-0.18983400
C	0.34580000	2.76350000	1.02150500
O	1.57549100	-2.18179600	0.81074200
H	-2.14867100	2.82482200	0.57047600
H	-2.13633700	2.62262100	-1.17549700
H	0.18492200	2.44465400	-1.10759800
H	1.42903900	2.63931900	1.09111200
H	-0.09933600	2.38486600	1.94860000
H	0.12154800	3.83444100	0.95058400
H	4.50629300	0.81707500	-0.84631600
C	3.48038400	-0.95560700	-0.01694600
H	4.26579300	-1.67044600	0.18106900
N	2.40899100	0.97241600	-0.51675100
H	2.24083500	1.72084700	-1.17479900
C	-3.13883800	-1.28563300	-0.02365200
C	-3.49014100	0.05663600	-0.06219200
C	-2.27756400	0.76156300	-0.09570300

C	-1.22013100	-0.15627600	-0.05422100
N	-1.77676700	-1.43034700	-0.02258600
H	-4.49803000	0.44879700	-0.06878800
C	-1.13332500	-2.72874400	-0.19438800
H	-1.80315700	-3.49719400	0.19995300
H	-0.95387700	-2.92809900	-1.25824500
H	-0.18361000	-2.74071300	0.33971000
H	-3.76803200	-2.16562400	-0.01264200

(2) Two transition state geometries at ground state for the two-stroke LDMRM DDPY.

EM-EP-TS

C	0.08395100	0.47829000	-0.10136500
C	1.39985000	0.08476800	-0.12081900
C	3.64857400	0.42389800	-0.38546600
C	2.13196200	-1.21729100	0.06495400
C	-1.74070900	2.15280900	-0.44073100
C	-0.22363000	2.00143100	-0.13393800
C	0.14162000	2.66417200	1.20877000
O	1.66549300	-2.31929600	0.37659600
H	-2.21273800	2.88699400	0.22419900
H	-1.90835200	2.50762100	-1.46720800
H	0.35332000	2.48635500	-0.93321200
H	1.19394900	2.51195100	1.46330600
H	-0.46780400	2.23916900	2.01428200
H	-0.05427500	3.74218900	1.16683000
H	4.53292600	1.01161800	-0.60243400
C	3.54093500	-0.90654100	-0.13665900
H	4.33946300	-1.62989500	-0.06016800
N	2.42791800	1.05282100	-0.30342400
H	2.25707700	1.91261500	-0.80362900
C	-3.16519900	-1.24491300	-0.04579700
C	-3.47938000	0.09240900	-0.23209100
C	-2.24930200	0.76550700	-0.24669200
C	-1.21393400	-0.16445600	-0.06944200
N	-1.81010900	-1.41693400	0.05073000
H	-4.47352600	0.50466200	-0.33858700
C	-1.22920300	-2.74834000	0.19967800
H	-1.32743500	-3.30772200	-0.73795600
H	-0.17320000	-2.66315300	0.45027300
H	-1.76519500	-3.28354600	0.98974000

H	-3.81601500	-2.10615300	0.02823600

ZM-ZP-TS

C	0.09249900	0.32821300	-0.05741000
C	1.30383700	-0.29850000	-0.06230300
C	2.91221600	-1.91077800	-0.11473500
C	2.65990100	0.35436400	-0.17574600
C	-1.49435500	2.15668700	-0.57149600
C	-0.02952800	1.86546700	-0.10745100
C	0.25348700	2.48746200	1.27284600
O	2.88721200	1.56696600	-0.23273600
H	-1.93914000	2.98621100	-0.00867800
H	-1.52092500	2.44486500	-1.63157200
H	0.70761700	2.26561000	-0.80685900
H	1.27347800	2.26395400	1.59188900
H	-0.45196700	2.11067500	2.02388200
H	0.14656900	3.57755000	1.22167200
H	3.27837300	-2.93043500	-0.09575900
C	3.61415500	-0.74934300	-0.20925200
H	4.68541700	-0.63869700	-0.29066000
N	1.55807100	-1.68158800	-0.05051500
H	0.87837800	-2.39825700	0.12478200
C	-3.31525500	-1.05549600	-0.06152800
C	-3.47945800	0.28664400	-0.35693000
C	-2.18605500	0.84887300	-0.34366300
C	-1.27015400	-0.15840800	-0.05531400
N	-1.97816300	-1.34039400	0.12078700
H	-4.42370600	0.77884100	-0.54667500
C	-1.49310900	-2.65339600	0.49780000
H	-0.97498500	-3.14871300	-0.33364100
H	-0.82886500	-2.58667500	1.36601200
H	-2.34467100	-3.27732700	0.77736900
H	-4.05170900	-1.84019600	0.04932300

8. Cartesian coordinates for several structures of two-stroke LDMRM DDPY optimized with the CAM-B3LYP/6-31G(d) method

Unit of the Cartesian coordinate below is angstrom.

(1) Four ground state local minima geometries for the two-stroke LDMRM DDPY.

ZP

C	0.09941200	0.35205800	-0.07303100
C	1.29785300	-0.26925400	-0.08529400
C	2.88147000	-1.87837100	-0.25965700
C	2.64422700	0.37523300	-0.09393300
C	-1.50820100	2.11034400	-0.67428900
C	-0.06057900	1.87525200	-0.14489700
C	0.13343700	2.50437400	1.23947200
O	2.88069300	1.57566000	-0.01554000
H	-1.98948300	2.95287200	-0.16700400
H	-1.50029800	2.34613300	-1.74551500
H	0.69433600	2.29461300	-0.81188600
H	1.14230400	2.31020600	1.60578000
H	-0.59548000	2.10491600	1.95314200
H	-0.00508300	3.58885500	1.18164500
H	3.24326000	-2.89235000	-0.38055500
C	3.59241300	-0.73402500	-0.19921600
H	4.66665300	-0.62785200	-0.21800500
N	1.52105200	-1.66887000	-0.11288600
H	0.88371400	-2.26093300	-0.62653100
C	-3.25411400	-1.10879800	-0.01590500
C	-3.45367900	0.20415200	-0.38799300
C	-2.17690200	0.79881400	-0.40708400
C	-1.25059500	-0.16613200	-0.06777400
N	-1.91840100	-1.34695700	0.18089800
H	-4.41050300	0.66039600	-0.59882600
C	-1.38701500	-2.60679300	0.65891000
H	-1.11569000	-3.27420000	-0.16802400
H	-0.50706100	-2.42974800	1.27701100
H	-2.14548100	-3.10744400	1.26346700
H	-3.97165800	-1.90108800	0.14768400

ZM

C	0.08645700	0.34053200	-0.04689000
C	1.28290300	-0.28939900	-0.01692600
C	2.87098100	-1.90525700	0.05925100
C	2.61992200	0.32930500	-0.26547900
C	-1.51649600	2.18529000	-0.42403900
C	-0.02539500	1.87079600	-0.10885400
C	0.40806500	2.50606600	1.21705400
O	2.85475500	1.51376300	-0.48008600
H	-1.90732600	2.96537900	0.23827300
H	-1.63841400	2.55347300	-1.44960000
H	0.64262500	2.24374200	-0.88764300
H	1.45390100	2.28120100	1.42865000
H	-0.21466500	2.14354900	2.04269900
H	0.30090300	3.59430200	1.16406300
H	3.24387200	-2.90949600	0.22122200
C	3.56582100	-0.78596100	-0.22582200
H	4.62984900	-0.69608700	-0.38490900
N	1.50573400	-1.68496100	0.11273600
H	0.96573700	-2.21208700	0.78246000
C	-3.30163600	-1.05060700	-0.07983500
C	-3.48067600	0.30256500	-0.26613300
C	-2.19169200	0.86716100	-0.23758400
C	-1.27575100	-0.14714900	-0.03383400
N	-1.96875000	-1.33970600	0.05427900
H	-4.42887600	0.80041200	-0.41056600
C	-1.46800800	-2.68068300	0.26332100
H	-0.69617300	-2.92452100	-0.46817200
H	-1.06892900	-2.80460200	1.27705500
H	-2.29534200	-3.38133600	0.14358900
H	-4.03106600	-1.84731600	-0.03092800

EP

C	0.10661600	0.52203800	-0.05695000
C	1.41051200	0.15340800	-0.04296400
C	3.66028900	0.45710100	-0.03922900
C	2.08420600	-1.16426100	-0.25524400
C	-1.70400300	2.01388100	-0.78335800
C	-0.28381400	2.00504000	-0.14907500
C	-0.30675100	2.66364900	1.23693800
O	1.54285400	-2.24559100	-0.47388000
H	-2.33412300	2.80260100	-0.35849100
H	-1.64313100	2.19776400	-1.86271500
H	0.43282000	2.53263700	-0.78676400

H	0.65616600	2.58652400	1.75338100
H	-1.05627500	2.17966600	1.87072100
H	-0.56160500	3.72492300	1.15541500
H	4.57490700	1.02895200	0.05870200
C	3.51106000	-0.87348900	-0.23050600
H	4.28960600	-1.60869900	-0.36541600
N	2.45464500	1.10953800	-0.00034200
H	2.34916000	1.99505000	0.46809000
C	-3.01736500	-1.37078900	-0.01594700
C	-3.37615300	-0.11519100	-0.46786200
C	-2.18995700	0.63394900	-0.47819900
C	-1.15075100	-0.18824100	-0.06781800
N	-1.67504000	-1.42922400	0.22631400
H	-4.37277800	0.19722100	-0.74552500
C	-1.04533800	-2.53107600	0.93543400
H	-1.67835700	-3.41354000	0.82582800
H	-0.06304600	-2.72265700	0.50755000
H	-0.95182600	-2.29542100	2.00104200
H	-3.63288100	-2.23860100	0.17610000

EM

C	0.07309300	0.48916400	-0.12687300
C	1.36842300	0.07335700	-0.15323800
C	3.60891000	0.31190100	-0.47856900
C	2.06349600	-1.19815800	0.22093900
C	-1.76605700	2.16511100	-0.27656100
C	-0.22538500	2.00466300	-0.18562800
C	0.32614100	2.72290800	1.05277100
O	1.57163000	-2.20251700	0.73003000
H	-2.13448200	2.84239000	0.50206500
H	-2.07522400	2.59521800	-1.23654700
H	0.23824800	2.43884800	-1.08109300
H	1.40323900	2.58374900	1.16008900
H	-0.15821500	2.33402200	1.95410200
H	0.11657900	3.79582700	0.99235700
H	4.50517400	0.83250100	-0.79311400
C	3.47604100	-0.95929900	-0.04185700
H	4.25813300	-1.68342700	0.12802200
N	2.41243700	0.98397400	-0.47040200
H	2.25135000	1.75154500	-1.10516200
C	-3.14217200	-1.25605900	-0.01960000
C	-3.47673200	0.08138100	-0.08407600
C	-2.25655700	0.76877000	-0.11850500

C	-1.21940600	-0.15440200	-0.05382300
N	-1.78820900	-1.41538600	-0.00498100
H	-4.47825200	0.48653300	-0.10613800
C	-1.16791100	-2.72366000	-0.13694200
H	-0.98447300	-2.95426700	-1.19193700
H	-0.22247000	-2.74011900	0.39970800
H	-1.85280700	-3.46862100	0.27255300
H	-3.78074600	-2.12823300	0.00431000

(2) Two transition state geometries at ground state for the two-stroke LDMRM DDPY.

EM-EP-TS

C	0.07986800	0.49120700	-0.11157600
C	1.37773900	0.08694700	-0.13510500
C	3.61752600	0.34320500	-0.45422800
C	2.07484400	-1.20385200	0.16405500
C	-1.75703900	2.15915500	-0.33566800
C	-0.22398900	2.00532200	-0.16415800
C	0.25941600	2.70252200	1.11401100
O	1.58155900	-2.23593000	0.60848300
H	-2.16787900	2.85533700	0.40403300
H	-2.01672000	2.56119200	-1.32222000
H	0.28394800	2.45696100	-1.02591600
H	1.32945700	2.55884700	1.27569200
H	-0.27263300	2.29903700	1.98139900
H	0.05513700	3.77686900	1.06115600
H	4.51321500	0.88230700	-0.73788900
C	3.48679300	-0.95125700	-0.09281900
H	4.26980700	-1.68349800	0.03212700
N	2.42088700	1.01408400	-0.40444800
H	2.25995600	1.81707600	-0.99338200
C	-3.14076100	-1.25430300	-0.04011300
C	-3.47148700	0.08051700	-0.14919600
C	-2.24956300	0.76501800	-0.16601400
C	-1.21433800	-0.15638100	-0.05379800
N	-1.78750400	-1.41481800	0.01485100
H	-4.47116700	0.48618000	-0.20949400
C	-1.17426500	-2.73322700	-0.00195500
H	-1.93825200	-3.46047800	0.27762100
H	-0.80428600	-2.97289800	-1.00345700
H	-0.33345900	-2.76950000	0.68241800

H	-3.78174000	-2.12409600	-0.00339500

ZM-ZP-TS

C	0.09689800	0.32901300	-0.06274300
C	1.29506900	-0.29751100	-0.06585600
C	2.90170700	-1.89823900	-0.11609600
C	2.64408500	0.35568100	-0.17245100
C	-1.48521200	2.14952900	-0.57040700
C	-0.02652800	1.85868200	-0.11200200
C	0.25407000	2.46938900	1.26581300
O	2.86393600	1.56207600	-0.22224900
H	-1.92653900	2.97573800	-0.00315800
H	-1.51638400	2.43821700	-1.62803700
H	0.71019300	2.26005600	-0.80910200
H	1.27319600	2.24485400	1.58305200
H	-0.45100700	2.08543200	2.01163700
H	0.14512200	3.55799300	1.22346100
H	3.27421500	-2.91498900	-0.09721300
C	3.60078000	-0.74314000	-0.20666800
H	4.67107000	-0.63013200	-0.28446700
N	1.55167600	-1.67916800	-0.05498200
H	0.87597700	-2.39651500	0.12498900
C	-3.30181300	-1.05092900	-0.05513300
C	-3.46742100	0.28367900	-0.34998900
C	-2.17487500	0.84401100	-0.34254100
C	-1.26860100	-0.15744200	-0.05806900
N	-1.97026600	-1.33399400	0.12169600
H	-4.41116600	0.77633900	-0.53589100
C	-1.48117800	-2.64141900	0.49239500
H	-0.96682900	-3.13226100	-0.34177900
H	-0.81483100	-2.57434400	1.35726600
H	-2.32880400	-3.26749800	0.77436000
H	-4.03744800	-1.83518800	0.05835300

9. Cartesian coordinates for several structures of two-stroke LDMRM DDPY optimized with the B3LYP-D3/6-31G+(d) method

Unit of the Cartesian coordinate below is angstrom.

(1) Four ground state local minima geometries for the two-stroke LDMRM DDPY.

ZP

C	0.09348500	0.35517700	-0.08209300
C	1.30720200	-0.26177900	-0.09829800
C	2.89626800	-1.87702400	-0.29191200
C	2.65730800	0.38430600	-0.07808900
C	-1.52708400	2.10711800	-0.69829100
C	-0.07560800	1.88292000	-0.14948100
C	0.09470900	2.50646400	1.24878900
O	2.89551300	1.59306400	0.03275600
H	-2.02212500	2.95217900	-0.20490500
H	-1.50553700	2.33059800	-1.77413600
H	0.68327000	2.31389600	-0.80744600
H	1.09350600	2.29782500	1.63938700
H	-0.65796800	2.10887700	1.94180400
H	-0.03024200	3.59465900	1.19196500
H	3.25700600	-2.88978500	-0.43202900
C	3.60759200	-0.72273400	-0.19548900
H	4.68326000	-0.61429500	-0.19597400
N	1.53092700	-1.66261300	-0.15532100
H	0.89086100	-2.25090600	-0.67323800
C	-3.25542200	-1.13355300	-0.00951300
C	-3.46523000	0.18545400	-0.39730100
C	-2.19088600	0.79055800	-0.42539100
C	-1.24746900	-0.17257800	-0.07905000
N	-1.91057600	-1.36060800	0.18580000
H	-4.42743100	0.63244300	-0.61038800
C	-1.36388800	-2.60309800	0.70999300
H	-1.07457800	-3.28924700	-0.09678300
H	-0.49185800	-2.39090700	1.33067200
H	-2.12169100	-3.09699300	1.32366100
H	-3.96890200	-1.92770600	0.16878300

ZM

C	-0.07782100	-0.34256100	-0.04584000
C	-1.28859600	0.28605800	-0.00874400
C	-2.87928400	1.91090500	0.11207200
C	-2.62842200	-0.32318800	-0.28987700
C	1.53698200	-2.19919900	-0.38781400
C	0.03209700	-1.87910300	-0.10893100
C	-0.44083300	-2.51993000	1.20908700
O	-2.86667300	-1.50762500	-0.55752800
H	1.91688700	-2.96683200	0.29819900
H	1.68179100	-2.58621100	-1.40564700
H	-0.61363500	-2.24920900	-0.91048500
H	-1.48647800	-2.27450400	1.40734500
H	0.17741000	-2.17438600	2.04805000
H	-0.35483000	-3.61157800	1.14697500
H	-3.24938800	2.91115600	0.30730300
C	-3.57447900	0.79102900	-0.21591900
H	-4.63820900	0.70557600	-0.38882700
N	-1.51064300	1.67970500	0.16234700
H	-0.96617900	2.18958300	0.84445100
C	3.31551900	1.05963300	-0.08235600
C	3.49837900	-0.30756800	-0.24204400
C	2.20866600	-0.87532700	-0.21137300
C	1.27862300	0.14799300	-0.03012400
N	1.97455100	1.34912600	0.03835800
H	4.44980300	-0.80599300	-0.37272800
C	1.46534400	2.70042000	0.20144500
H	0.69640600	2.91436200	-0.54421200
H	1.05933600	2.85161600	1.20997800
H	2.29090200	3.40238400	0.06473900
H	4.04514800	1.85825200	-0.04733400

EP

C	0.09530500	0.53370700	-0.06073800
C	1.41200400	0.16602800	-0.03364300
C	3.67009800	0.47415800	0.00229800
C	2.08749400	-1.14591300	-0.28783300
C	-1.74389700	1.99219000	-0.81308000
C	-0.31676500	2.01536100	-0.17092000
C	-0.35570700	2.69296500	1.21376600
O	1.53905300	-2.22230400	-0.56991900
H	-2.39156900	2.77657600	-0.40233000
H	-1.67803600	2.16044500	-1.89685000
H	0.39273500	2.55000000	-0.81403500

H	0.60571200	2.62833100	1.73774500
H	-1.10744000	2.20849100	1.84803300
H	-0.61848300	3.75306000	1.11612800
H	4.58278400	1.04529100	0.12697800
C	3.51578200	-0.85960700	-0.23215300
H	4.29502300	-1.59244000	-0.38689600
N	2.45556200	1.12317000	0.04577300
H	2.34492300	2.00379300	0.52630900
C	-2.99403300	-1.42005200	0.01887200
C	-3.38205900	-0.16831400	-0.45736500
C	-2.20938800	0.60543800	-0.49030600
C	-1.14203300	-0.19683400	-0.07190600
N	-1.64255200	-1.44817400	0.25018400
H	-4.38869300	0.11877700	-0.73162400
C	-0.97127100	-2.51563900	0.98599900
H	-1.62647500	-3.39016900	0.99686900
H	-0.02793500	-2.75755600	0.49694200
H	-0.78473800	-2.19769900	2.01914500
H	-3.59447600	-2.29436800	0.23329200

EM

C	0.05365500	0.53222000	-0.15201200
C	1.35009700	0.08888500	-0.18408700
C	3.60074400	0.21323300	-0.52156900
C	1.98900400	-1.15797600	0.34069800
C	-1.83922700	2.15992900	-0.11943800
C	-0.28108000	2.04182100	-0.22578900
C	0.41336600	2.84213700	0.89150000
O	1.43151100	-2.06500300	0.97431800
H	-2.11829200	2.75546900	0.75990100
H	-2.27393900	2.66661300	-0.99106600
H	0.04423200	2.43896500	-1.19950000
H	1.50176900	2.75061800	0.85088700
H	0.08205800	2.47838400	1.87149900
H	0.14878400	3.90382500	0.81462000
H	4.51531300	0.66354800	-0.88983100
C	3.41411000	-1.00373500	0.06091200
H	4.17181400	-1.72968700	0.32041900
N	2.42064400	0.92555500	-0.59366000
H	2.28643400	1.63724200	-1.29947300
C	-3.07910100	-1.34463700	-0.05514300
C	-3.47475200	-0.00963900	-0.00454000
C	-2.28601700	0.73800400	-0.01943100

C	-1.20355400	-0.14938600	-0.04661600
N	-1.71231500	-1.44008000	-0.09214500
H	-4.49632000	0.34638000	0.01668200
C	-1.00732400	-2.70130900	-0.30515000
H	-1.72451700	-3.42571900	-0.69994600
H	-0.20712600	-2.55576800	-1.03310200
H	-0.55774000	-3.06303800	0.61815700
H	-3.68238400	-2.24242100	-0.08915700

(2) Two transition state geometries at ground state for the two-stroke LDMRM DDPY.

EM-EP-TS

C	0.08462200	0.50032300	-0.11794600
C	1.39560700	0.10104400	-0.14418300
C	3.64971200	0.37790600	-0.40443300
C	2.08735500	-1.20707500	0.09807800
C	-1.75885200	2.15997400	-0.39940600
C	-0.22996500	2.01818300	-0.14516000
C	0.17608000	2.67725000	1.18808800
O	1.56383100	-2.26940100	0.45571800
H	-2.21598200	2.87777000	0.29355700
H	-1.96631900	2.52431900	-1.41506600
H	0.32067700	2.50491600	-0.96205700
H	1.23433300	2.51741700	1.41405300
H	-0.41475700	2.25114500	2.00777700
H	-0.01582800	3.75671300	1.15361400
H	4.55053600	0.93394300	-0.63775500
C	3.50720300	-0.94240000	-0.10498300
H	4.28888000	-1.68085000	0.00368700
N	2.44297300	1.04044000	-0.35592000
H	2.29794500	1.89348400	-0.87689900
C	-3.13816000	-1.26526400	-0.05670000
C	-3.47275300	0.07613400	-0.19804100
C	-2.25039600	0.76488300	-0.21041000
C	-1.20204800	-0.15758000	-0.06871700
N	-1.77760000	-1.42214700	0.01606100
H	-4.47372300	0.47647200	-0.28860800
C	-1.16920500	-2.74535200	0.14860000
H	-1.97576500	-3.48136900	0.10339400
H	-0.46068000	-2.92638400	-0.65868600

H	-0.63134000	-2.83498700	1.09090800
H	-3.78144600	-2.13359600	-0.00336400

ZM-ZP-TS

C	0.09094000	0.33135300	-0.06700800
C	1.30351000	-0.29350000	-0.07084300
C	2.91150000	-1.90759200	-0.12312600
C	2.65836800	0.35950900	-0.16905700
C	-1.50045300	2.15654100	-0.57364500
C	-0.03487700	1.86675300	-0.11058000
C	0.24314900	2.47204700	1.27838500
O	2.88638800	1.57530900	-0.21674800
H	-1.94904800	2.98152000	-0.00681800
H	-1.52728800	2.44487100	-1.63366800
H	0.70009600	2.27558000	-0.80796700
H	1.25476100	2.22520200	1.60937200
H	-0.48122000	2.09727800	2.01340900
H	0.15662800	3.56464600	1.23632400
H	3.27880300	-2.92712900	-0.10784600
C	3.61393600	-0.74212200	-0.20105800
H	4.68600300	-0.62833300	-0.27458000
N	1.55566600	-1.67906700	-0.07754700
H	0.88114700	-2.39299500	0.12827100
C	-3.31053000	-1.06412900	-0.05820500
C	-3.48000400	0.28101400	-0.35577700
C	-2.18761400	0.84626200	-0.34885300
C	-1.26757100	-0.16036600	-0.06459700
N	-1.97131700	-1.34411200	0.11764200
H	-4.42674100	0.77070900	-0.54208300
C	-1.47624700	-2.64876100	0.51505200
H	-0.95482200	-3.14828400	-0.31110700
H	-0.81584700	-2.55950300	1.38432500
H	-2.32249900	-3.27656000	0.80300000
H	-4.04615800	-1.84927500	0.05855600

10. References

[S1] W. Thiel, MNDO program, version 6.1, Max-Planck-Institut fur Kohlenforschung: Mulheim, 2007.

[S2] Frisch, M. J.; Trucks, G. W.; Schlegel, H. B.; Scuseria, G. E.; Robb, M. A.; Cheeseman, J. R.; Scalmani, G.; Barone, V.; Mennucci, B.; Petersson, G. A.; Nakatsuji, H.; Caricato, M.; Li, X.; Hratchian, H. P.; Izmaylov, A. F.; Bloino, J.; Zheng, G.; Sonnenberg, J. L.; Hada, M.; Ehara, M.; Toyota, K.; Fukuda, R.; Hasegawa, J.; Ishida, M.; Nakajima, T.; Honda, Y.; Kitao, O.; Nakai, N.; Vreven, T.; Montgomery Jr, J. A.; Peralta, J. E.; Ogliaro, F.; Bearpark, M.; Heyd, J. J.; Brothers, E.; Kudin, K. N.; Staroverov, V. N.; Keith, T.; Kobayashi, R.; Normand, J.; Raghavachari, K.; Rendell, A.; Burant, J. C.; Iyengar, S. S.; Tomasi, J.; Cossi, M.; Rega, N.; Millam, J. M.; Klene, M.; Knox, J. E.; Cross, J. B.; Bakken, V.; Adamo, C.; Jaramillo, J.; Gomperts, R.; Stratmann, R. E.; Yazyev, O.; Austin, A. J.; Cammi, R.; Pomelli, C.; Ochterski, J. W.; Martin, R. L.; Morokuma, R.; Zakrzewski, V. G.; Voth, G. A.; Salvador, P.; Dannenberg, J. J.; Dapprich, S.; Daniels, A. D.; Farkas, O.; Foresman, J. B.; Ortiz, J. V.; Cioslowski, J.; Fox, D. J.; Gaussian 09, Revision D.01, Gaussian, Inc., Wallingford CT, 2009.
